# Supplementary material for: Highly selective urea electrooxidation coupled with efficient hydrogen evolution
Source: Nat Commun. 2024 Jul 14;15:5918. doi: 10.1038/s41467-024-50343-8 (PMC11247087; doi:10.1038/s41467-024-50343-8)
Supplement: Supplementary file 1 — Supplementary information [file 41467_2024_50343_MOESM1_ESM.pdf]

Supplementary Information

Highly Selective Urea Electrooxidation Coupled with Efficient Hydrogen Evolution

Guangming Zhan[1,2], Lufa Hu[1,2], Hao Li[1]*, Jie Dai[1], Long Zhao[1], Qian Zheng[1], Xingyue Zou[1], Yanbiao Shi[1], Jiaxian Wang[1], Wei Hou[1], Yancai Yao[1]*, Lizhi Zhang[1]*

[1]School of Environmental Science and Engineering, Shanghai Jiao Tong University, Shanghai 200240, P. R. China. [2]These authors contributed equally: Guangming Zhan, Lufa Hu. *e-mail: hao_li@sjtu.edu.cn; yyancai@sjtu.edu.cn; zhanglizhi@sjtu.edu.cn

Supplementary Figures

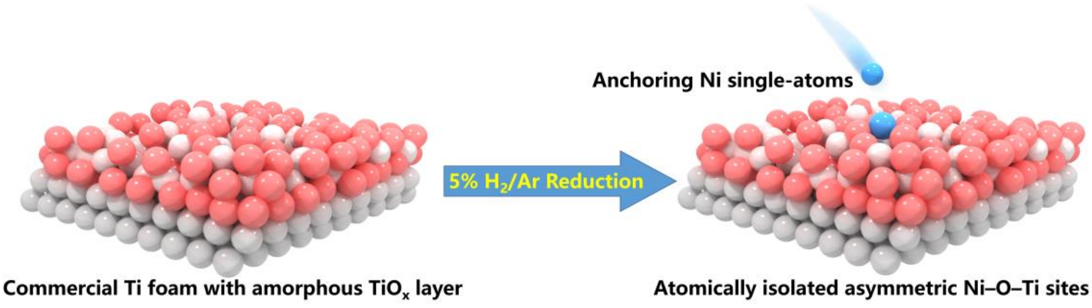

Supplementary Figure 1. Schematic illustration of the synthetic procedures of atomically isolated asymmetric Ni–O–Ti sites.

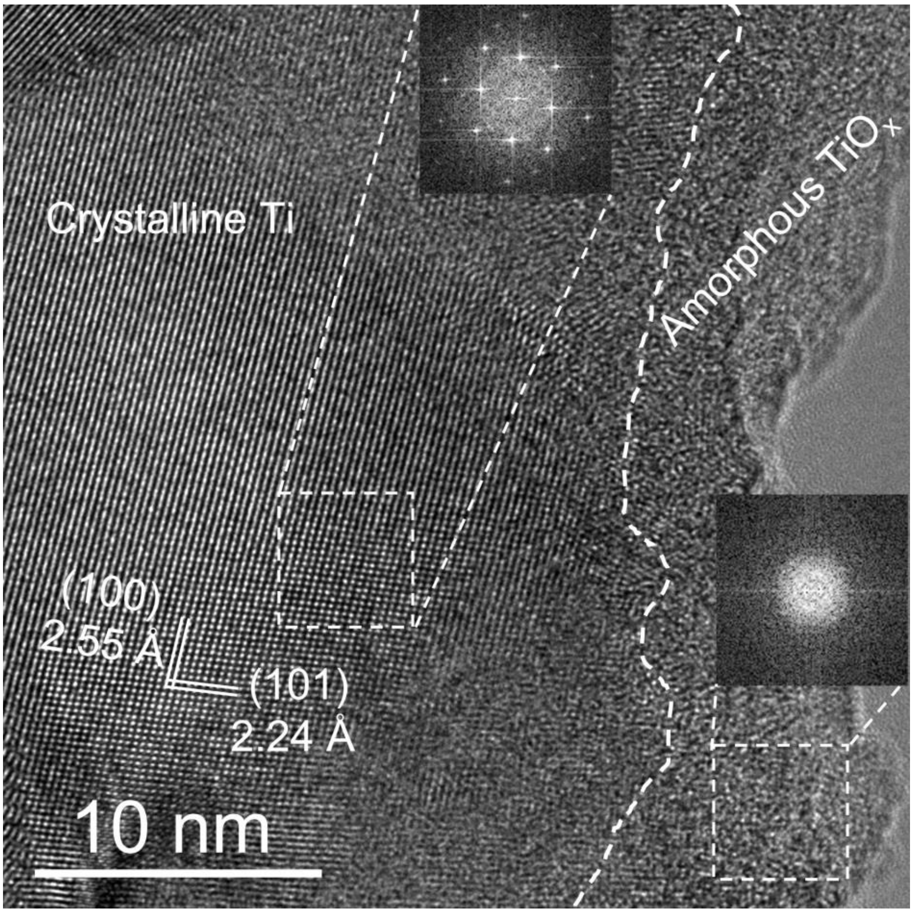

Supplementary Figure 2. The high-resolution transmission electron microscopy (HRTEM) image of Ti foam. A distinct boundary was observed between the interior crystalline Ti and surface amorphous TiO_x layer according to the selected area electron diffraction SAED pattern. Moreover, the interior crystalline Ti showed 2.55 Å and 2.24 Å lattice fingers corresponding to the (100) and (101) atomic planes, respectively, consistent with our previous work[1].

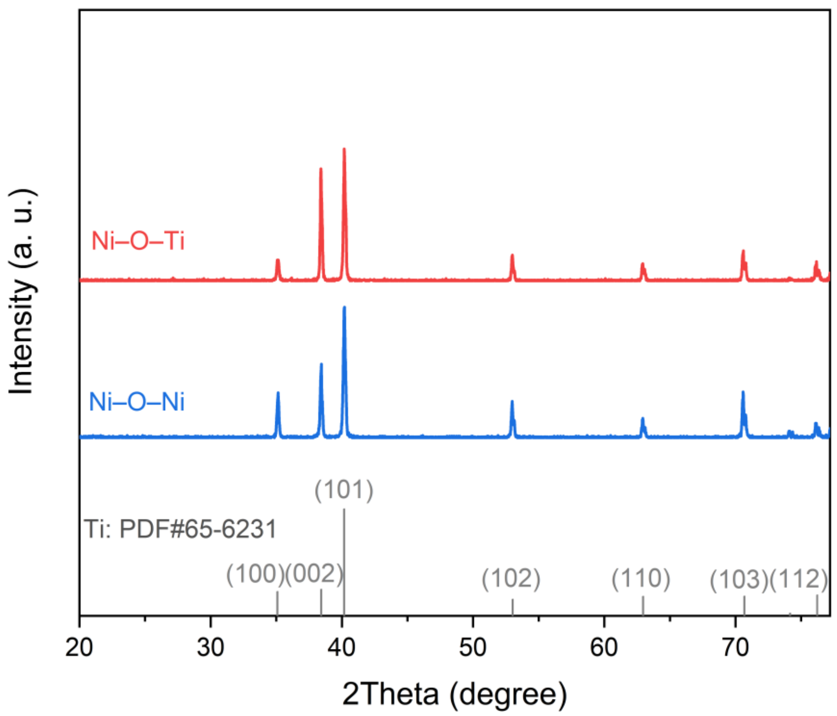

Supplementary Figure 3. X-ray diffraction (XRD) patterns of atomically isolated asymmetric Ni–O–Ti and connected symmetric Ni–O–Ni sites on Ti foam.

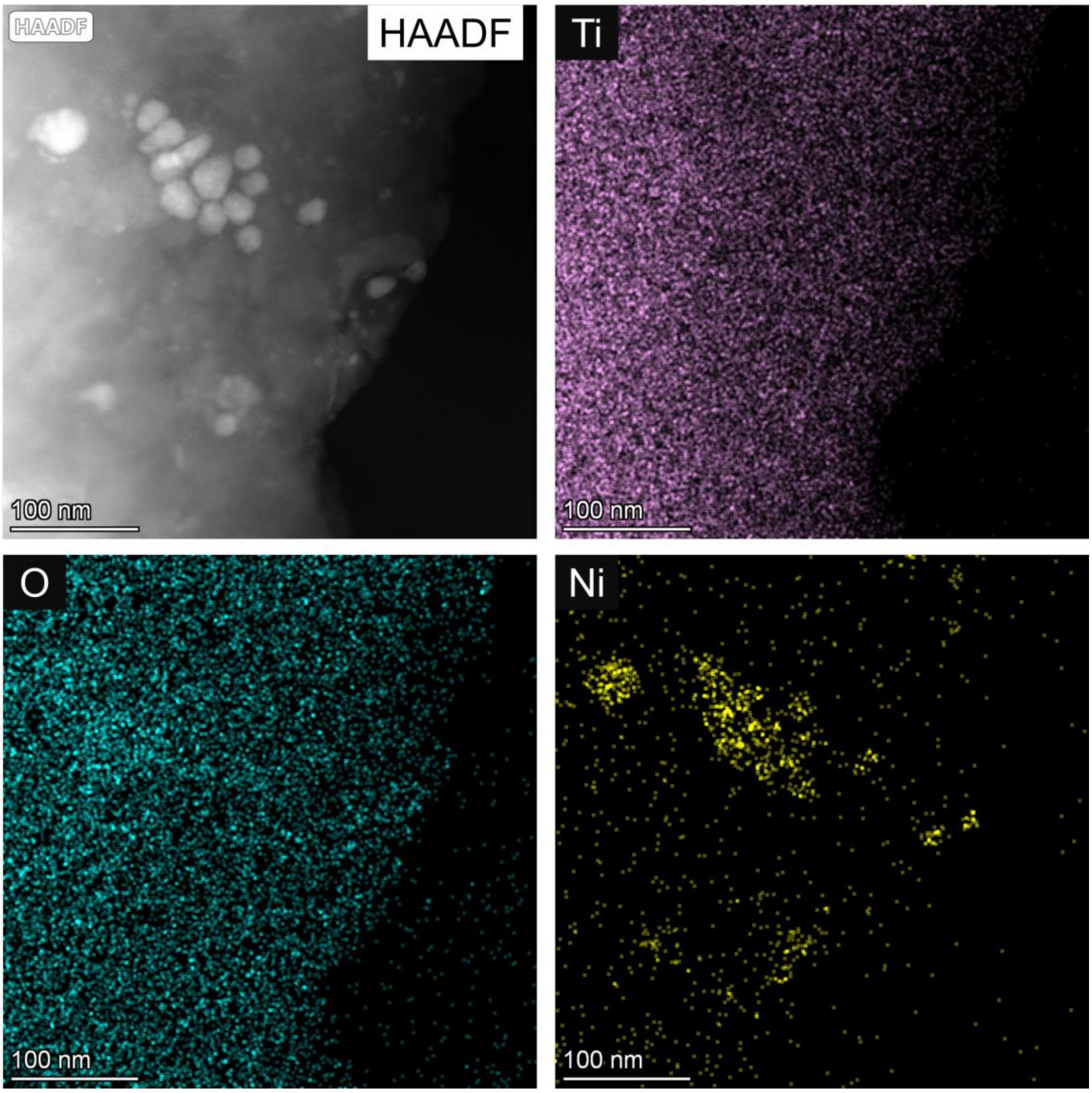

Supplementary Figure 4. STEM elemental mapping of connected symmetric Ni–O–Ni sites on Ti foam. Discernible Ni nanoparticles were formed on the Ti foam with increasing Ni loading.

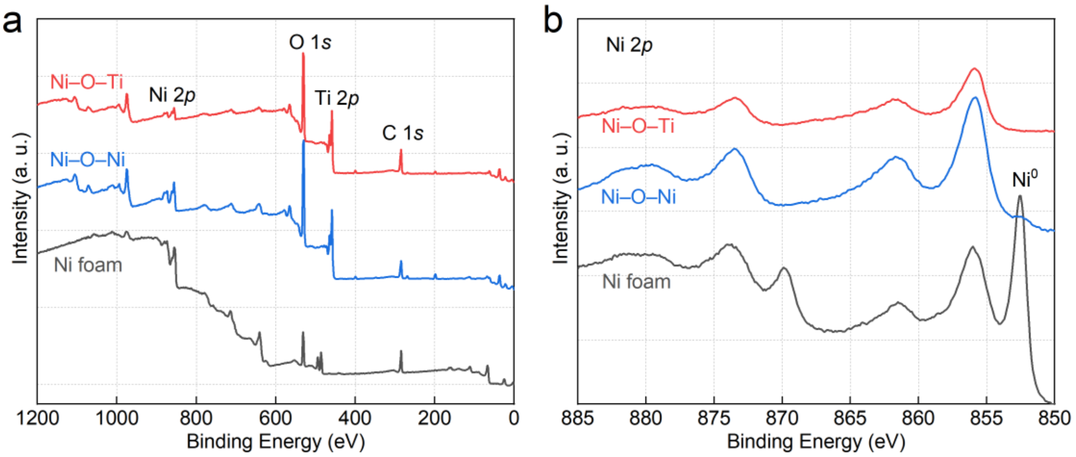

Supplementary Figure 5. (a) Total and (b) high-resolution Ni 2*p* XPS spectrum of atomically isolated asymmetric Ni–O–Ti and connected symmetric Ni–O–Ni sites on Ti foam. Based on the Ni 2*p* XPS of Ni foam, the binding energy of 851.7 eV of connected symmetric Ni–O–Ni sites on Ti foam can be indexed to the metallic Ni^0, indicating a more metallic state of connected symmetric Ni–O–Ni sites than that of atomically isolated asymmetric Ni–O–Ti sites.

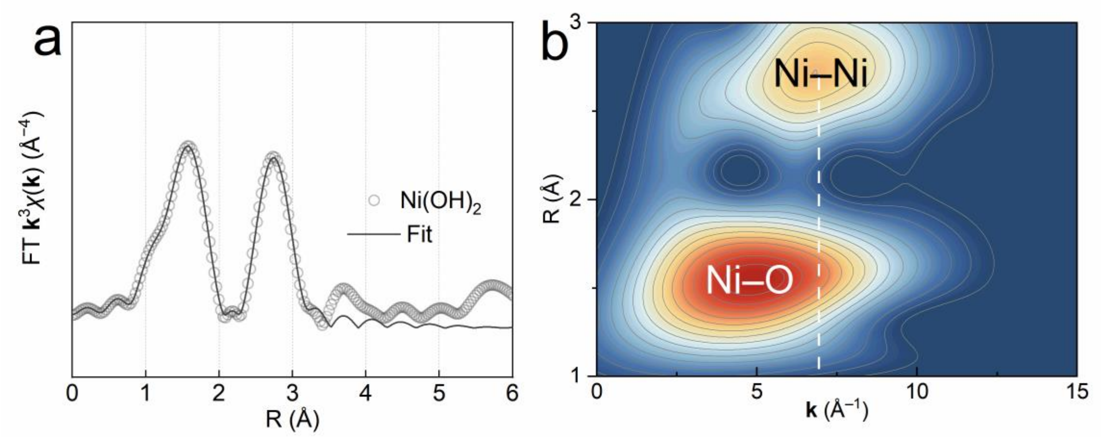

Supplementary Figure 6. (a) FT-EXAFS and (b) WT-EXAFS spectra of $Ni(OH)_2$.

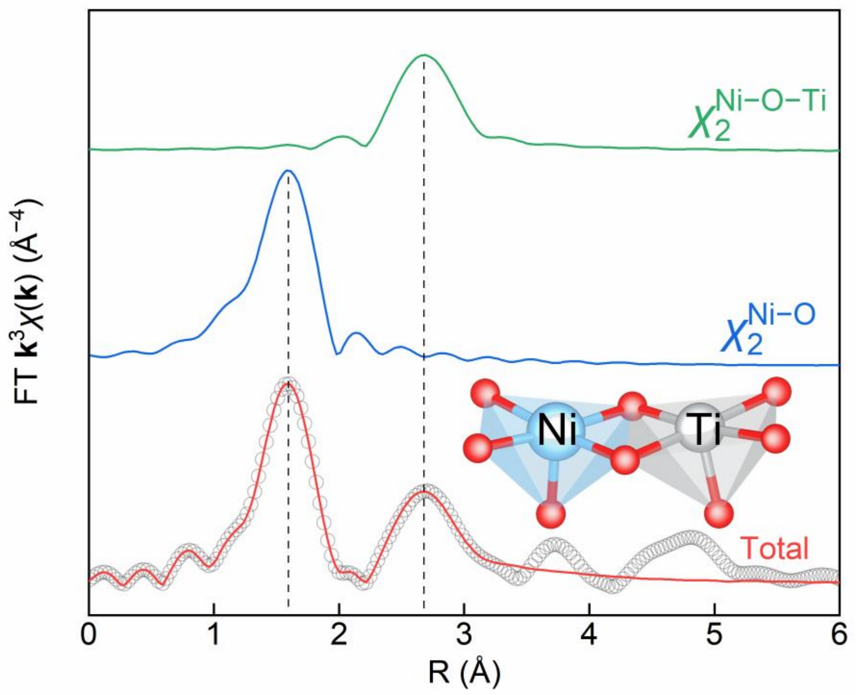

Supplementary Figure 7. The specific contribution of Ni–O–Ti and Ni–O shells coordination in FT-EXAFS for Ni–O–Ti sites. The inset displayed the structure of a Ni–O–Ti moiety derived from the EXAFS result, where the blue, red, and grey spheres represent Ni, O and Ti, respectively.

Curves from top to bottom were the Ni–O–Ti and Ni–O two-body backscattering signals χ_2 included in the fit and the total signal (red line) superimposed on the experimental signal (black dots). The measured and calculated spectra agreed well. The best-fitting analyses revealed that the main peak was originated from Ni–O first shell coordination and the minor peak was well interpreted as the Ni–O–Ti contribution.

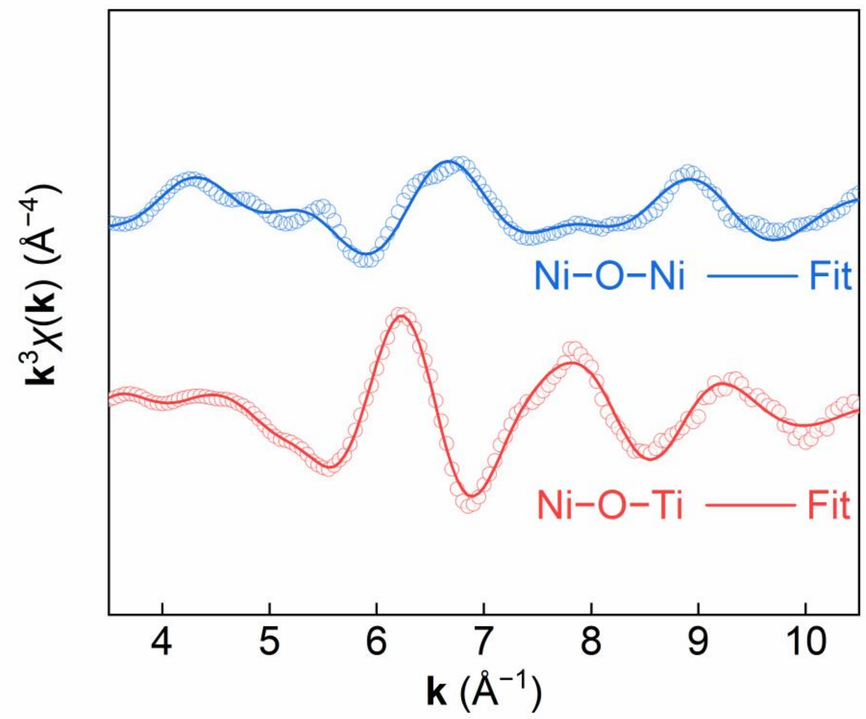

Supplementary Figure 8. EXAFS oscillations at **k**^{3}-weighted Ni K-edge and fitted curves of asymmetric Ni–O–Ti and symmetric Ni–O–Ni sites. The fitting curve of **k-**space was in good agreement with the original data, thus confirming the reasonableness of EXAFS fitting results.

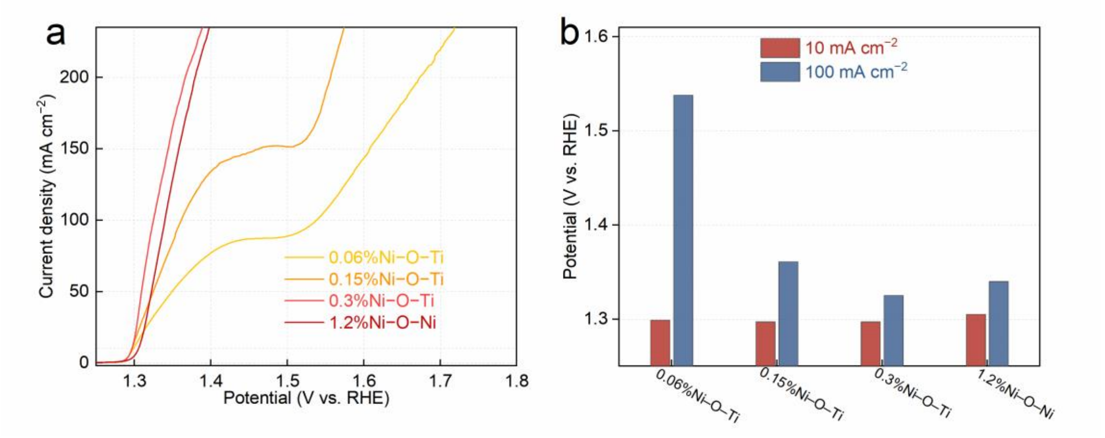

Supplementary Figure 9. (a) The LSV curves of Ti foam with different Ni loading weight percentages in 1.0 M KOH + 0.33 M urea. (b) The corresponding potentials of Ti foam with different Ni loading percentages at current density of 10 mA cm^{-2} and 100 mA cm^{-2}, respectively. The UOR performance of 0.06%Ni–O–Ti, 0.15%Ni–O–Ti, and 0.3%Ni–O–Ti enhanced with the increase of Ni loading and atomically isolated asymmetric Ni–O–Ti sites. After the aggregation of Ni atoms of 1.2%Ni–O–Ni into Ni nanoparticles exposed connected symmetric Ni–O–Ni sites, the UOR performance slightly decreased.

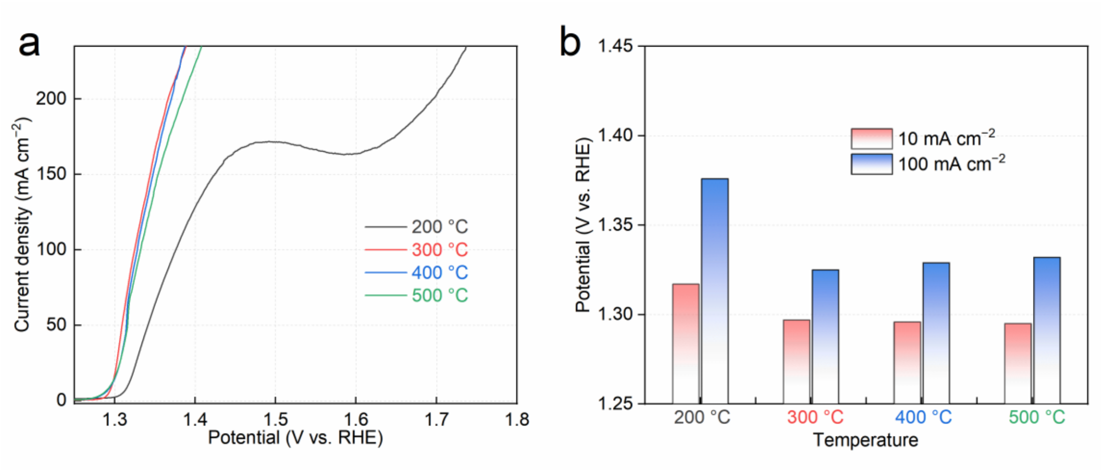

Supplementary Figure 10. (a) The LSV curves of Ti foam with atomically isolated asymmetric Ni–O–Ti sites under different preparation temperature of 200 ^{o}C, 300 ^{o}C, 400 ^{o}C, and 500 ^{o}C in 1.0 M KOH + 0.33 M urea. (b) The corresponding potentials of Ni–O–Ti sites with different preparation temperature at current density of 10 mA cm^{-2} and 100 mA cm^{-2}, respectively.

We adjusted the preparation temperatures to 200 ^{o}C, 300 ^{o}C, 400 ^{o}C, and 500 ^{o}C to construct the asymmetric Ni–O–Ti sites without changing other conditions. The activity of Ni–O–Ti sites increased when increasing the preparation temperature from 200 ^{o}C to 300 ^{o}C, and then slightly decreased with the temperature increasing from 300 ^{o}C to 500 ^{o}C. Specifically, the UOR potential at a current density of 10 mA cm^{-2} was 1.32 V_{RHE} at the preparation temperature of 200 ^{o}C, and decreased to 1.30 V_{RHE} when the temperature increased to 300 ^{o}C, 400 ^{o}C, and 500 ^{o}C. For the current density up to 100 mA cm^{-2}, the UOR potentials for the electrodes prepared at temperature of 200 ^{o}C, 300 ^{o}C, 400 ^{o}C, and 500 ^{o}C were 1.38, 1.32, 1.33, and 1.33 V_{RHE}, respectively.

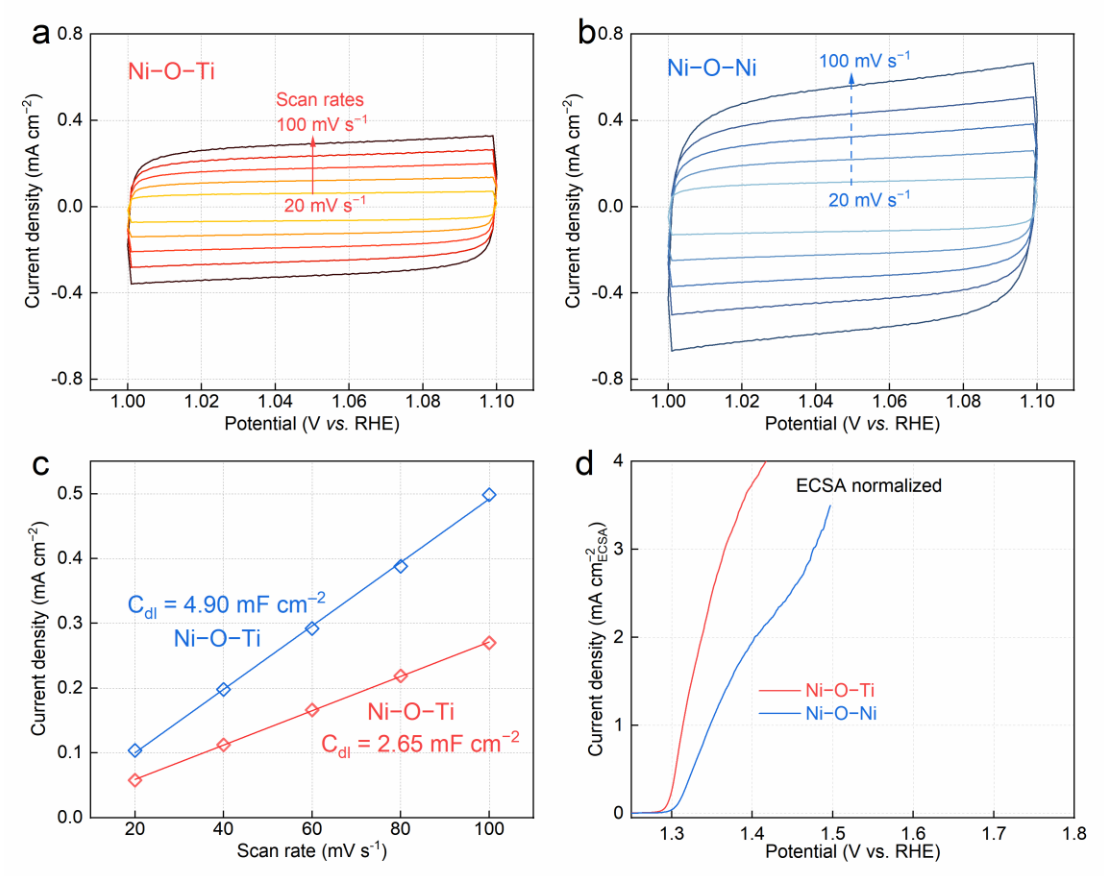

Supplementary Figure 11. CV curves recorded in a range of 1.00–1.10 V_{RHE} without Faraday current at different scan rates of 20, 40, 60, 80, 100 mV s^{-1} for (a) asymmetric Ni–O–Ti and (b) symmetric Ni–O–Ni sites. (c) Half of current density differences ($\Delta j/2$) at a fixed potential of 1.05 V_{RHE} plotted against scan rates derived from CV curves, and (d) the loaded normalized LSV of UOR on asymmetric Ni–O–Ti and symmetric Ni–O–Ni sites.

The electrochemical active surface area (ECSA) of asymmetric Ni–O–Ti and symmetric Ni–O–Ni sites were estimated by electrochemical double-layer capacitance (C_{dl}) measurements. ECSA was calculated according to the equation:

$$ECSA = C_{dl}/C_s$$

The C_{dl} is the fitting slope of $\Delta j/2$ against the scan rate. C_s is the specific capacitance of sample or the capacitance of an atomically smooth planar surface of the material per unit area under identical electrolyte conditions. To estimate the surface area, we used general specific capacitances of C_s = 0.040 mF cm^{-2} in 1.0 M KOH according to typical reported values[2]. As shown in Supplementary Fig. 11 a–c, the Ti foam with Ni–O–Ti sites had smaller C_{dl} value of 2.65 mF cm^{-2} than that of Ni–O–Ni sites (4.90 mF cm^{-2}). Therefore, the ECSA of Ni–O–Ti and Ni–O–Ni were 66.25 and 122.5 cm^2, respectively. We further normalized UOR activity using ECSA, and found that asymmetric Ni–O–Ti sites had a much higher intrinsic UOR activity than symmetric Ni–O–Ni sites (Supplementary Fig. 11d).

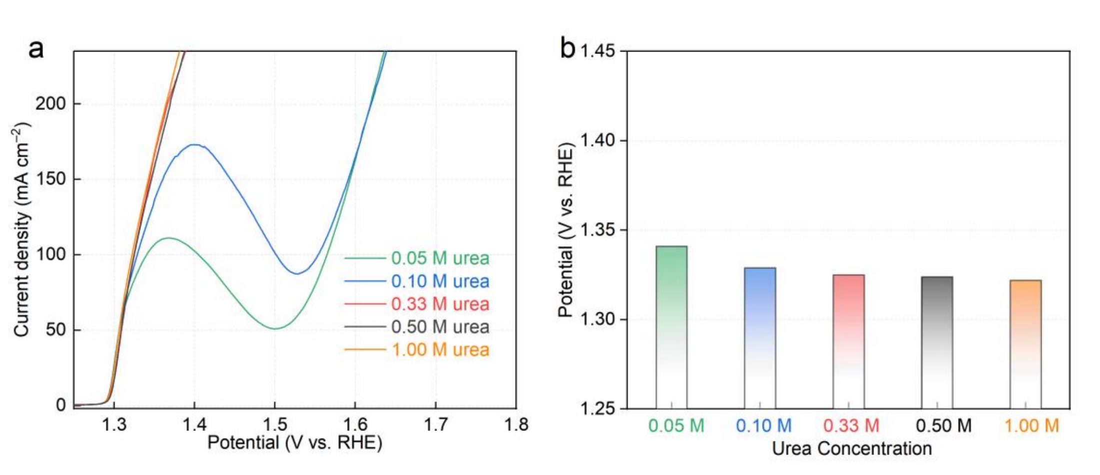

Supplementary Figure 12. (a) The LSV curves of atomically isolated asymmetric Ni–O–Ti in 1.0 M KOH containing different urea concentrations. (b) The corresponding potentials of Ni–O–Ti for achieving a current density of 100 mA cm^{-2} at different urea concentrations.

We measured the UOR activities of Ni–O–Ti sites by LSV measurements in 1.0 M KOH containing different urea concentrations. The current density of UOR gradually increased with increasing urea concentration up to 0.33 M, and then kept unchanged at higher concentration. Specifically, potentials of 1.34 and 1.33 V_{RHE} were respectively required to achieve a current density of 100 mA cm^{-2} at the urea concentrations of 0.05 and 0.10 M, and potential of 1.32 V_{RHE} was required at the urea concentrations of 0.33, 0.50, and 1.00 M.

The electrolytes were prepared with 1.0 M KOH combined with varying concentrations of urea: 0.05 M, 0.10 M, 0.33 M, 0.50 M, and 1.00 M. The respective mixtures were composed of 56.106 g of KOH with 3.003 g of urea, 56.106 g of KOH with 6.006 g of urea, 56.106 g of KOH with 20.020 g of urea, 56.106 g of KOH with 30.030 g of urea, and 56.106 g of KOH with 60.060 g of urea. Each solution was then diluted to a volume of 1.0 L using ultrapure water. Following preparation, the electrolytes were stored at room temperature and protected from light exposure.

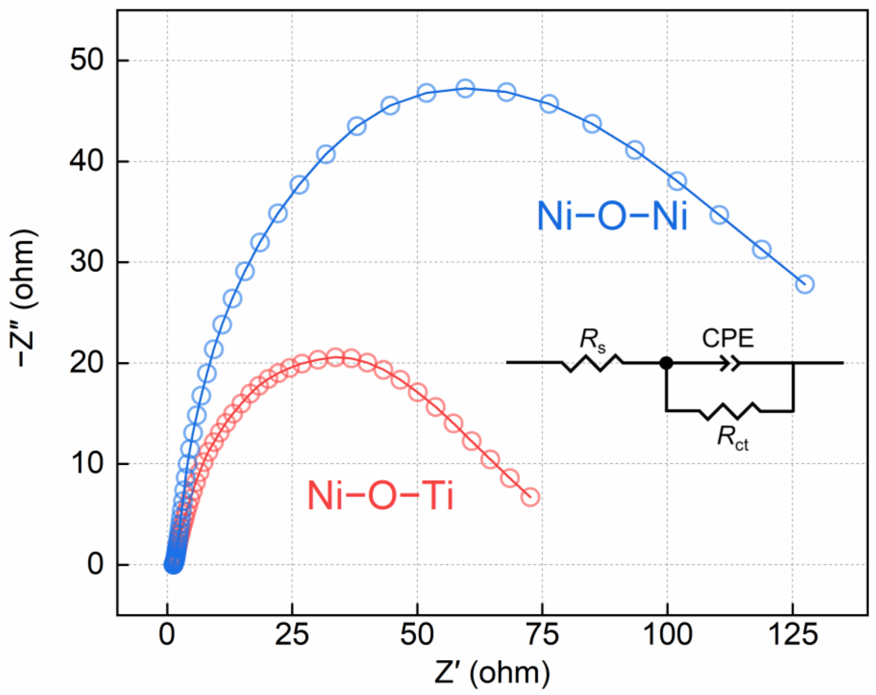

Supplementary Figure 13. The electrochemical impedance spectroscopy (EIS) spectra of symmetric Ni–O–Ni and asymmetric Ni–O–Ti sites at 1.30 V_{RHE} performed at a frequency range from 100 kHz to 0.1 Hz in 1.0 M KOH. Inset shows the equivalent electric circuit. The EIS plots were fitted using simplified Randles circuit model for obtaining the series resistance (R_s), capacitance of the double layer (C_d) and charge-transfer resistance (R_{ct}). The experimental results revealed that R_{ct} of Ni–O–Ti (73 Ω) was much smaller than that of Ni–O–Ni (138 Ω), indicating a favorable charge transfer kinetics of Ni–O–Ti.

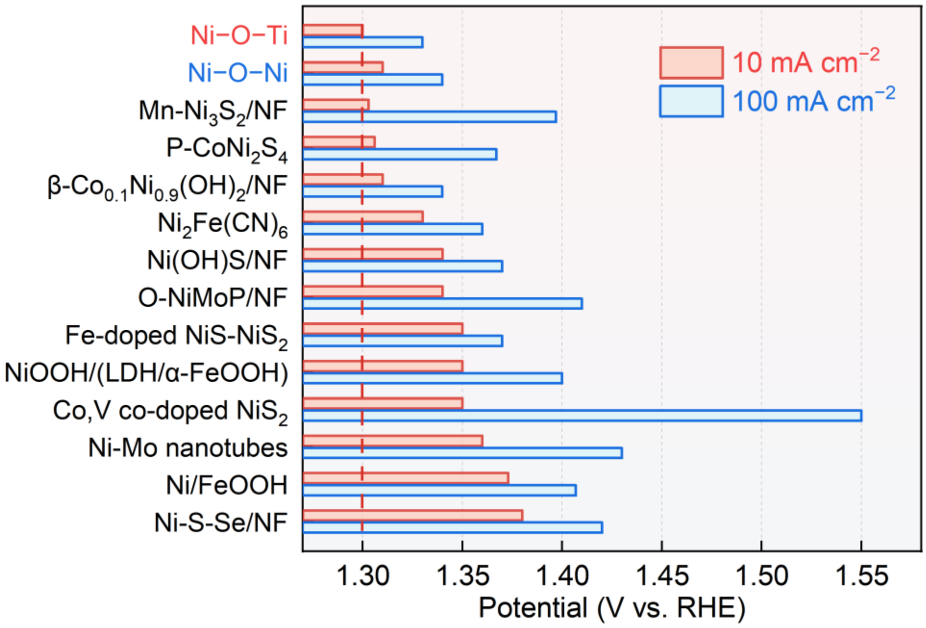

Supplementary Figure 14. Comparison of UOR potentials among the asymmetric Ni–O–Ti sites and reported Ni-based catalysts.

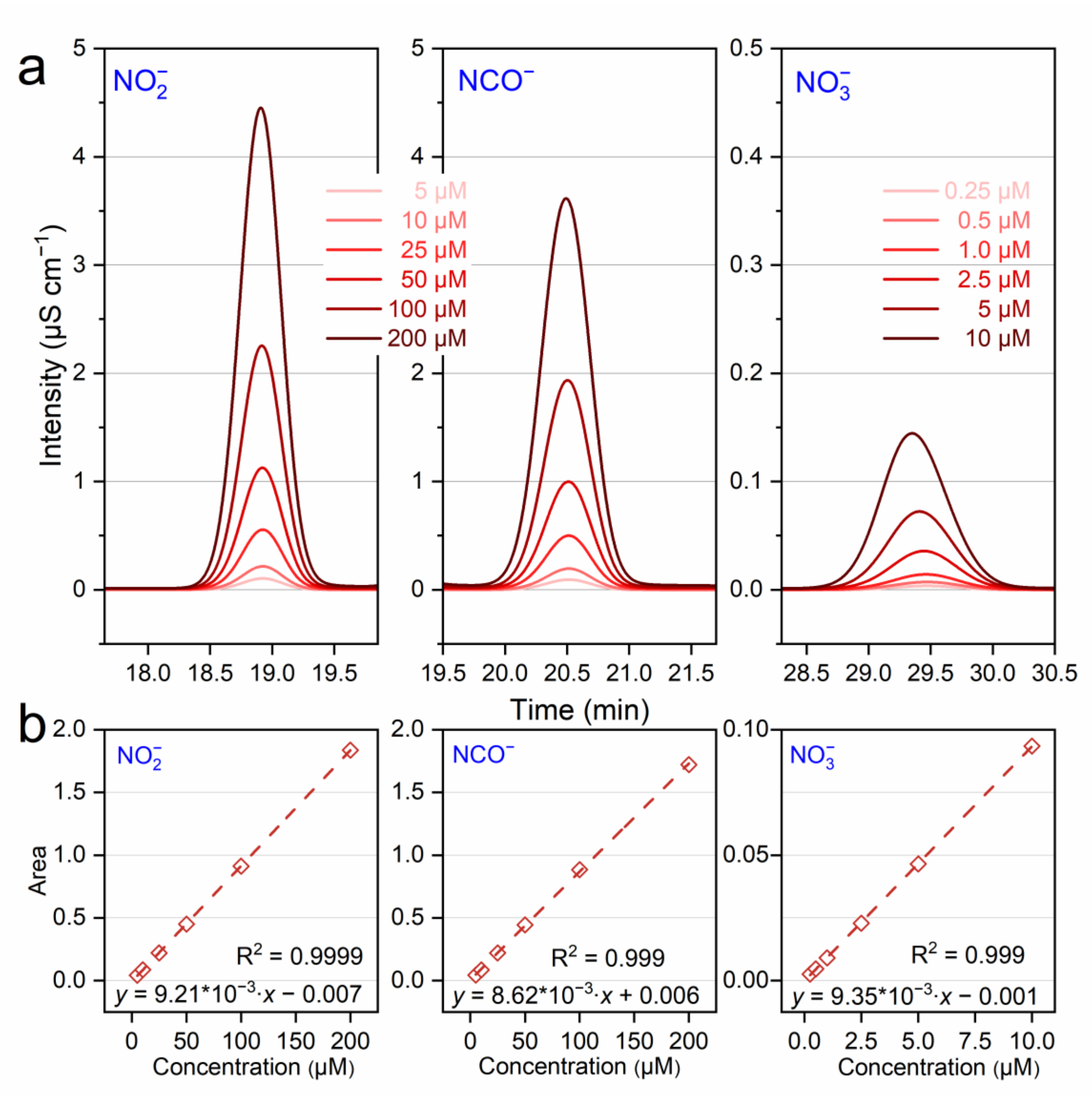

Supplementary Figure 15. Absolute calibration of Ion chromatograms using KNO_2 (5.0–200.0 μM), KNCO (5.0–200.0 μM), and KNO_3 (0.25–10.0 μM) solutions of known concentration as standards. (a) Ion chromatogram curves of various NO_2^-, NCO^-, and NO_3^- concentrations in 1.0 M KOH solution, respectively. (b) The standard curve for determination produced NO_2^-, NCO^-, and NO_3^-. The peak area of NO_2^-, NCO^-, and NO_3^- at 18.8 min, 20.5 min, and 29.4 min was measured by Ion chromatogram, respectively, and the fitting curve shows good linear relation of peak area with the concentration of NO_2^-, NCO^-, and NO_3^- solutions.

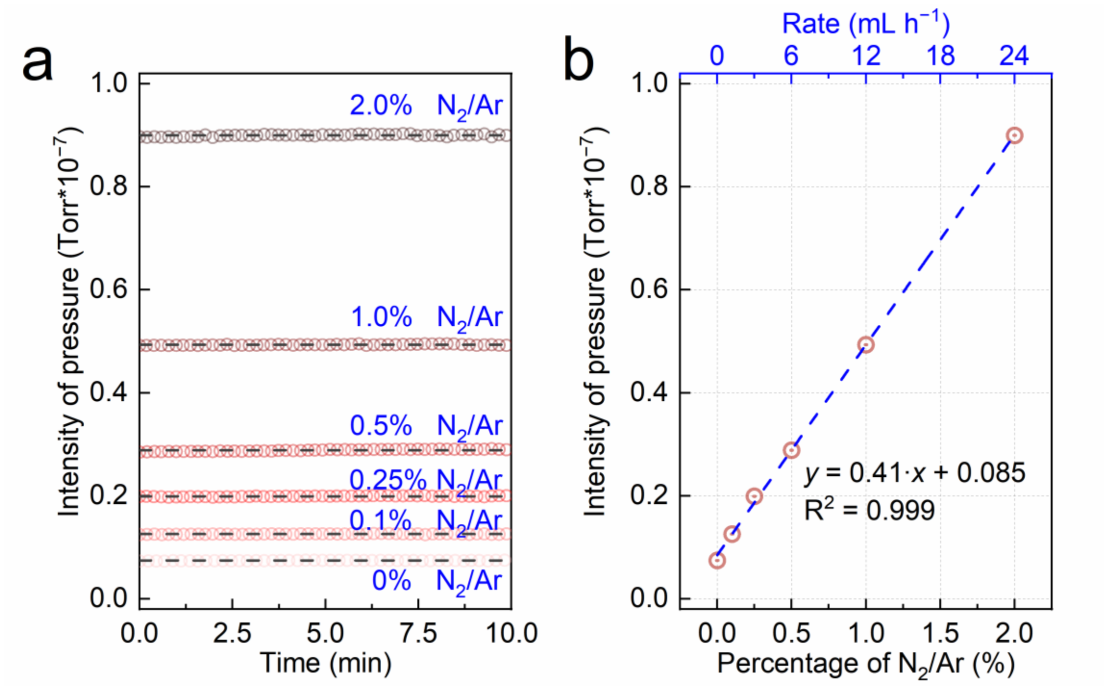

Supplementary Figure 16. The absolute calibration of on-line mass spectrometry (MS) using N_2/Ar mixture of known concentration (0%, 0.1%, 0.25%, 0.5%, 1.0%, and 2.0%) as standards. (a) on-line MS signal of various concentrations N_2/Ar mixture. (b) The standard curve used for determination produced N_2. The fitting curve shows good linear relationship between the on-line MS signal intensity and N_2 concentration ($y = 0.41*x + 0.085$, $R^2 = 0.999$) and the corresponding N_2 production rate.

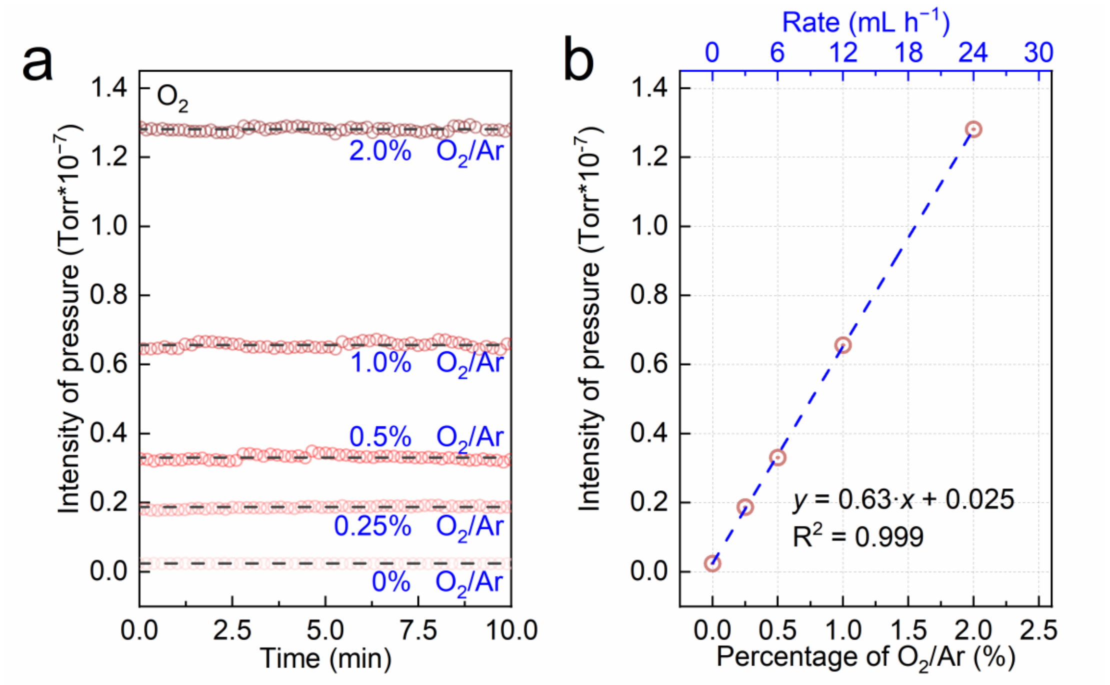

Supplementary Figure 17. The absolute calibration of on-line mass spectrometry (MS) using O_2/Ar mixture of known concentration (0%, 0.25%, 0.5%, 1.0%, and 2.0%) as standards. (a) on-line MS signal of various concentrations O_2/Ar mixture. (b) The standard curve used for determination produced O_2. The fitting curve shows good linear relationship between the on-line MS signal intensity and O_2 concentration ($y = 0.63*x + 0.025$, $R^2 = 0.999$) and the corresponding O_2 production rate.

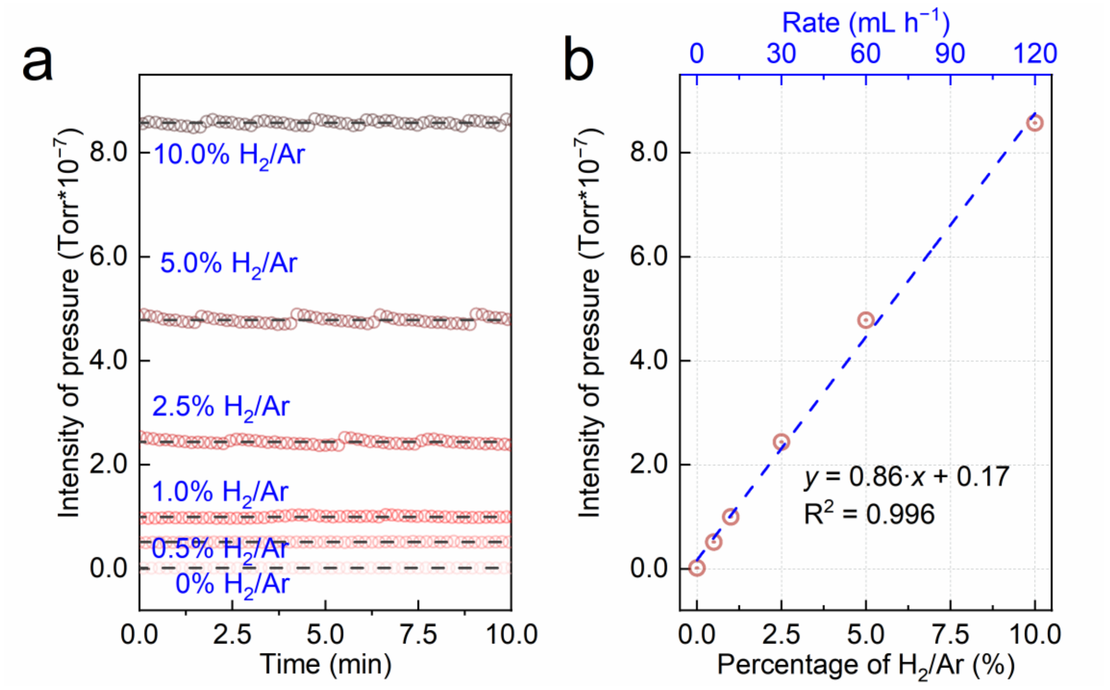

Supplementary Figure 18. The absolute calibration of on-line mass spectrometry (MS) using H_2/Ar mixture of known concentration (0%, 0.5%, 1.0%, 2.5%, 5.0%, and 10.0%) as standards. (a) on-line MS signal of various concentrations H_2/Ar mixture. (b) The standard curve used for determination produced H_2. The fitting curve shows good linear relationship between the on-line MS signal intensity and H_2 concentration (y = 0.86*x + 0.17, R^2 = 0.996) and the corresponding H_2 production rate.

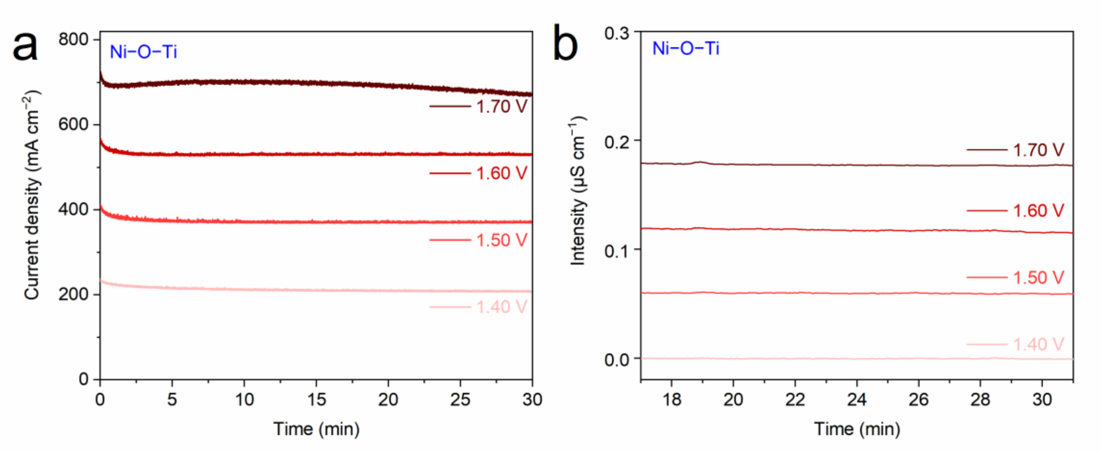

Supplementary Figure 19. (a) The *i*–t curve and (b) original data of Ion chromatograms for atomically isolated Ni–O–Ti sites after 30 min UOR process in 1.0 M KOH + 0.33 M urea solution under different applied potentials of 1.40, 1.50, 1.60 and 1.70 V_{RHE}, respectively.

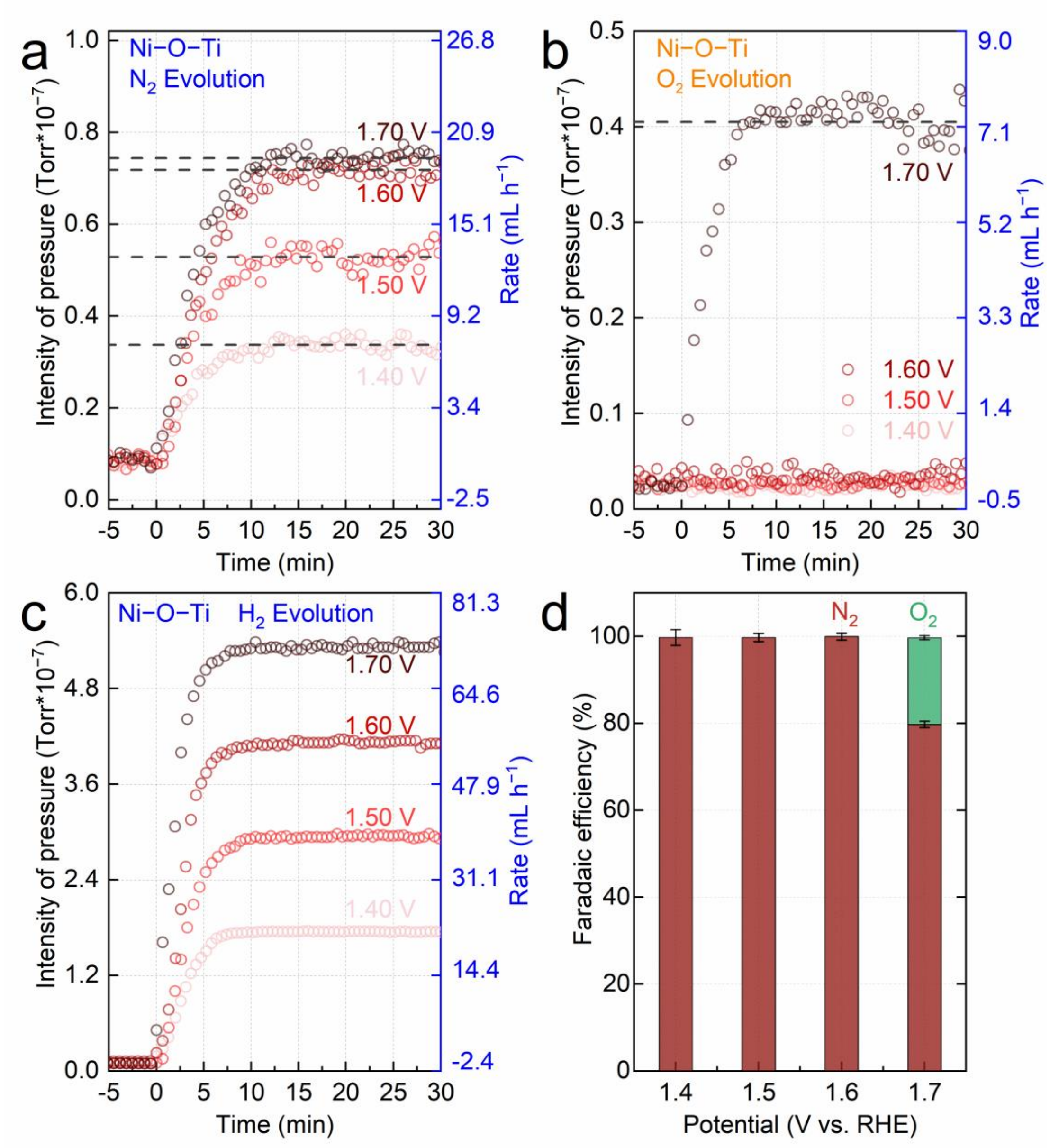

Supplementary Figure 20. The original data of on-line MS for (a) N_2 evolution, (b) O_2 evolution from anode chamber, (c) H_2 evolution and from cathode chamber, and (d) corresponding faradaic efficiencies of N_2 and O_2 during UOR on atomically isolated Ni–O–Ti sites in 1.0 M KOH + 0.33 M urea solution under different applied potentials of 1.40, 1.50, 1.60 and 1.70 V_{RHE}. Error bars represent the standard deviations derived from three distinct samples.

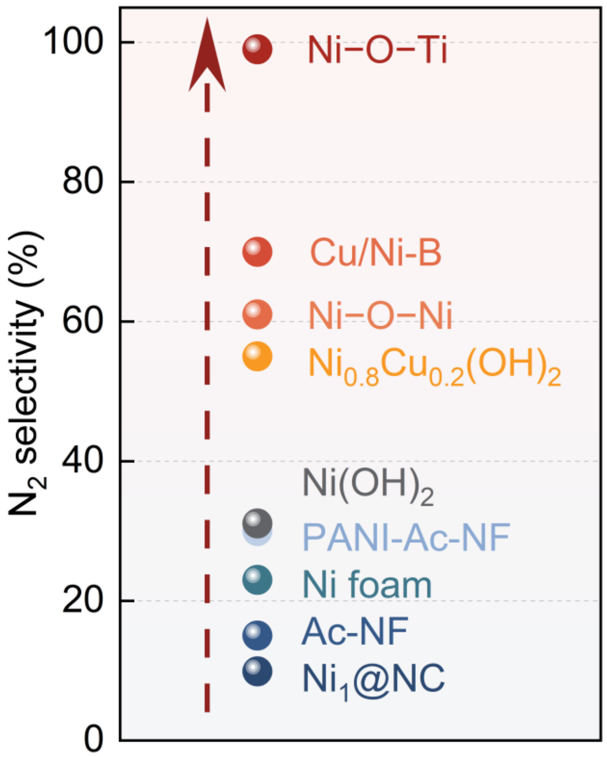

Supplementary Figure 21. The comparison of UOR selectivity of the catalysts prepared in this work and reported Ni-based catalysts.

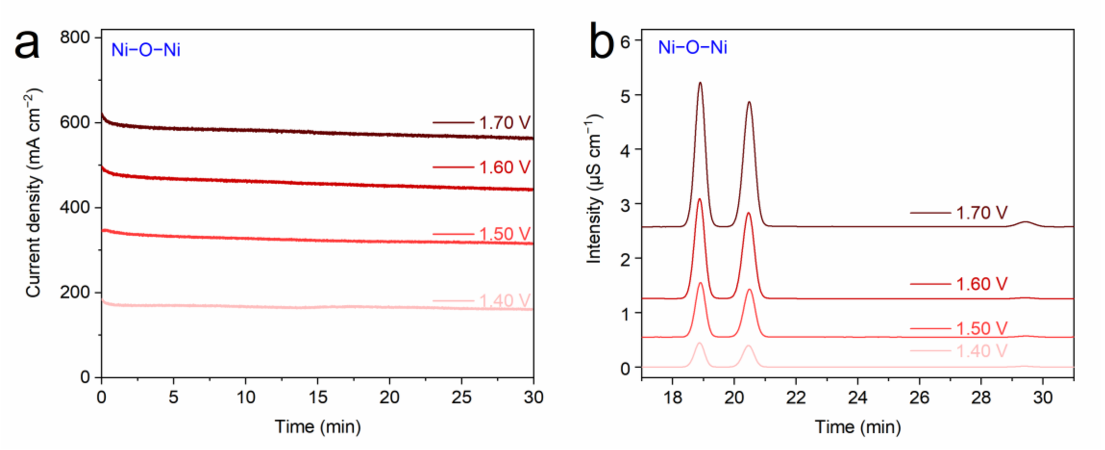

Supplementary Figure 22. (a) The *i*–t curve and (b) original data of Ion chromatograms for connected symmetric Ni–O–Ni sites after 30 min UOR process in 1.0 M KOH + 0.33 M urea solution under different applied potentials of 1.40, 1.50, 1.60 and 1.70 V_{RHE}, respectively.

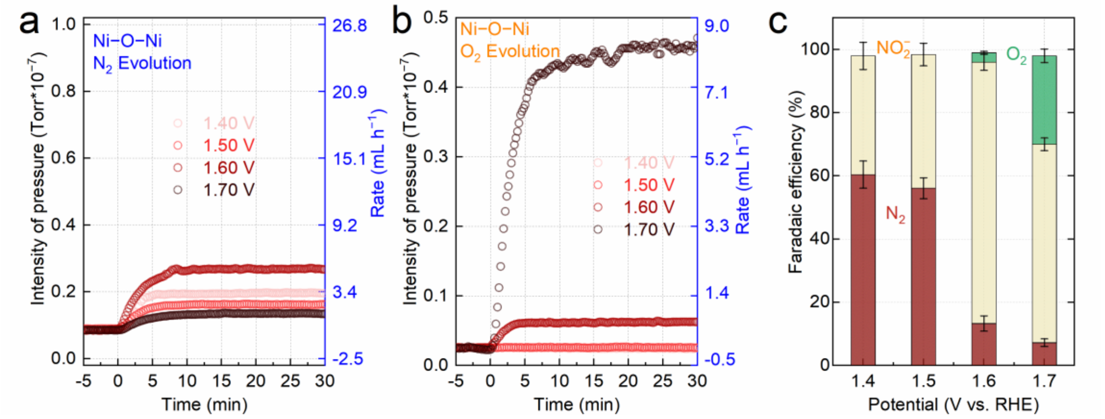

Supplementary Figure 23. The original data of on-line MS for (a) N_2 evolution, (b) O_2 evolution from anode chamber, and (c) corresponding faradaic efficiencies of N_2, NO_2^-, and O_2 during UOR on connected symmetric Ni–O–Ni sites in 1.0 M KOH + 0.33 M urea solution under different applied potentials of 1.40, 1.50, 1.60 and 1.70 V_{RHE}. Error bars represent the standard deviations derived from three distinct samples.

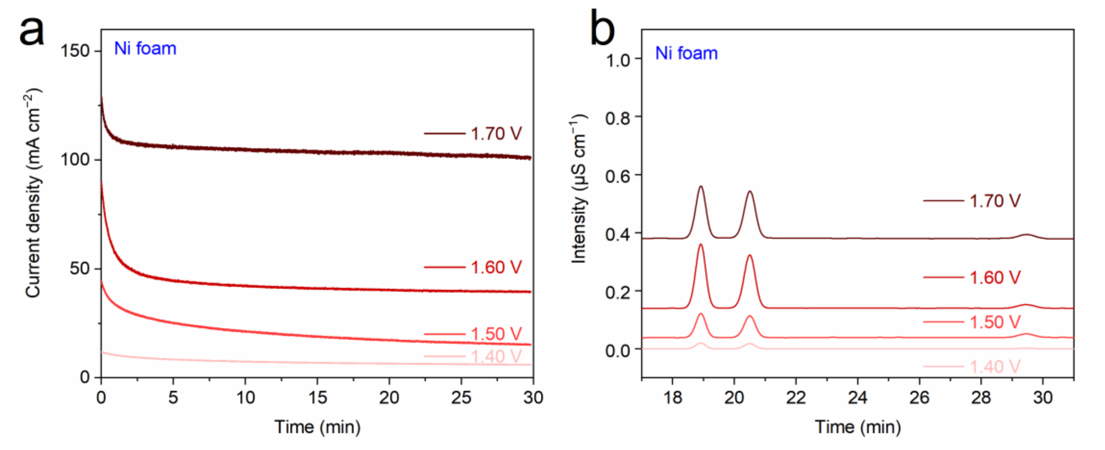

Supplementary Figure 24. (a) The *i*–t curve and (b) original data of Ion chromatograms for Ni foam after 30 min UOR process in 1.0 M KOH + 0.33 M urea solution under different applied potentials of 1.40, 1.50, 1.60 and 1.70 V_{RHE}, respectively.

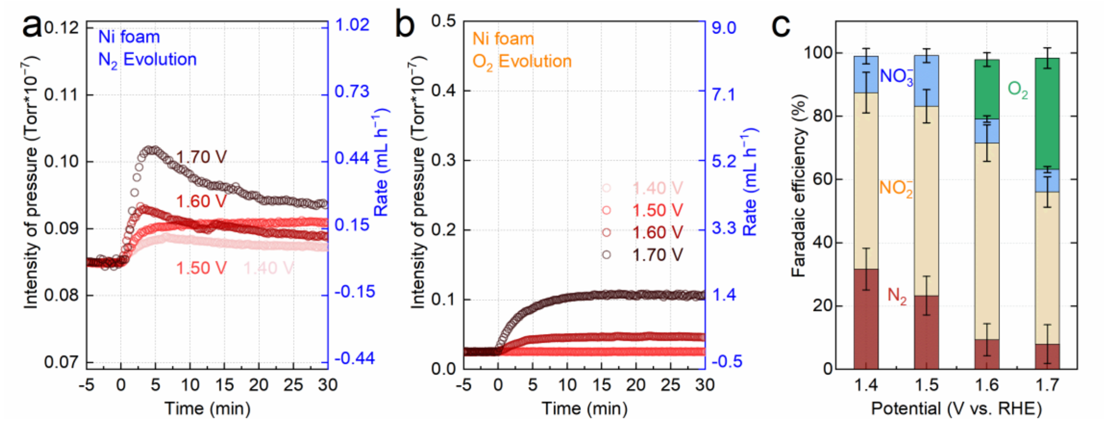

Supplementary Figure 25. The original data of on-line MS for (a) N_2 evolution, (b) O_2 evolution from anode chamber, and (c) corresponding faradaic efficiencies of N_2, NO_2^-, NO_3^-, and O_2 during UOR on Ni foam in 1.0 M KOH + 0.33 M urea solution under different applied potentials of 1.40, 1.50, 1.60 and 1.70 V_{RHE}. Error bars represent the standard deviations derived from three distinct samples.

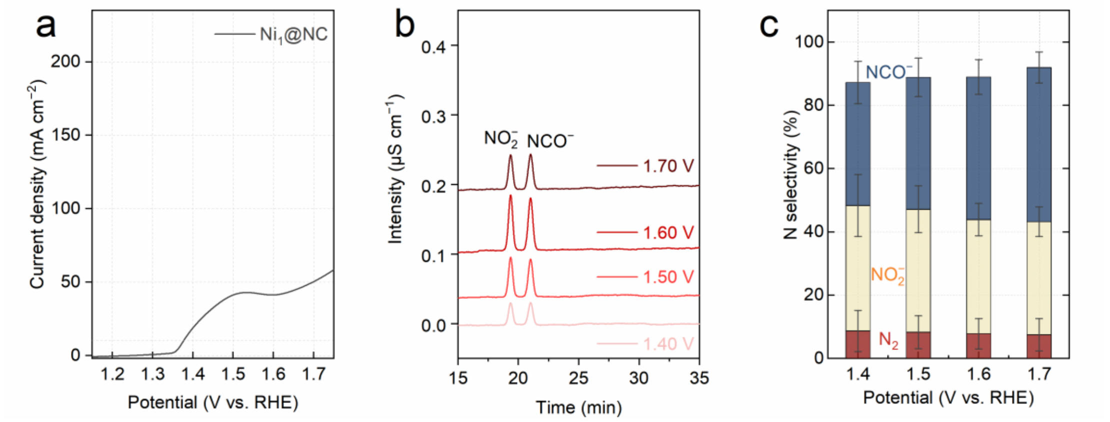

Supplementary Figure 26. (a) LSV curves of atomically isolated Ni sites on Ni_1@NC in 1.0 M KOH + 0.33 M urea. The original data of (b) ion chromatograms after 30 min UOR process and (c) corresponding N selectivities of urea for N_2, NO_2^-, and NO_3^- during UOR for Ni_1@NC in 1.0 M KOH + 0.33 M urea solution under different applied potentials of 1.40, 1.50, 1.60 and 1.70 V_{RHE}, respectively. Error bars represent the standard deviations derived from . samples.

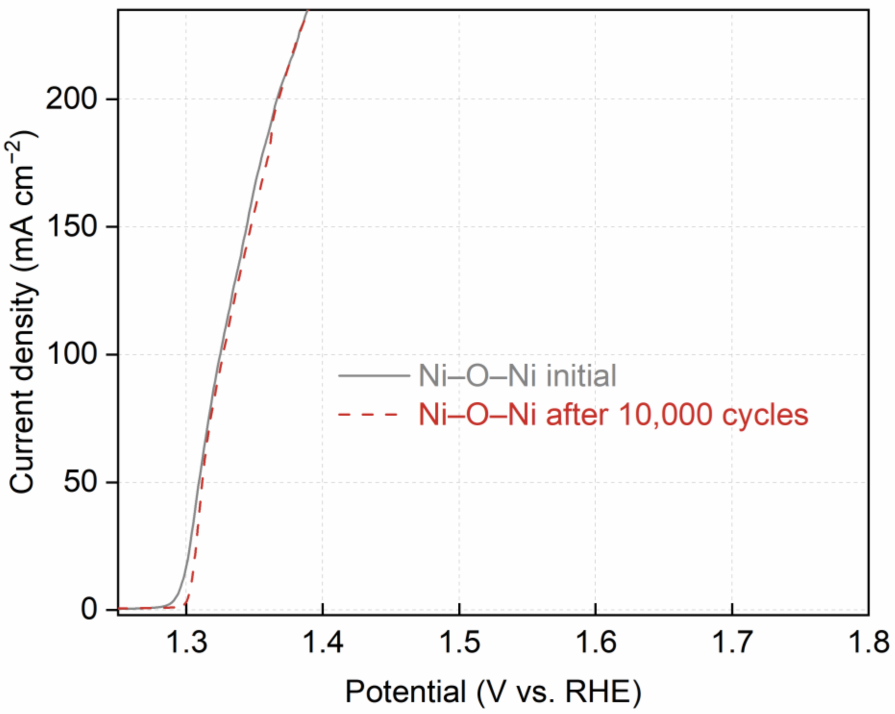

Supplementary Figure 27. LSV curves of atomically isolated Ni–O–Ti sites after 10,000 cycles in 1.0 M KOH + 0.33 M urea.

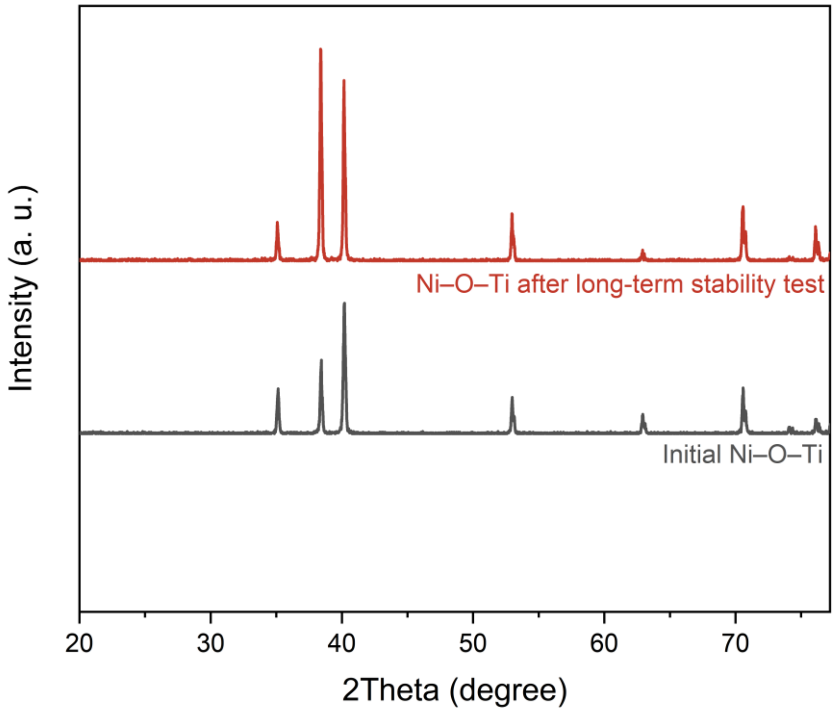

Supplementary Figure 28. XRD of atomically isolated Ni–O–Ti sites after long-time stability test.

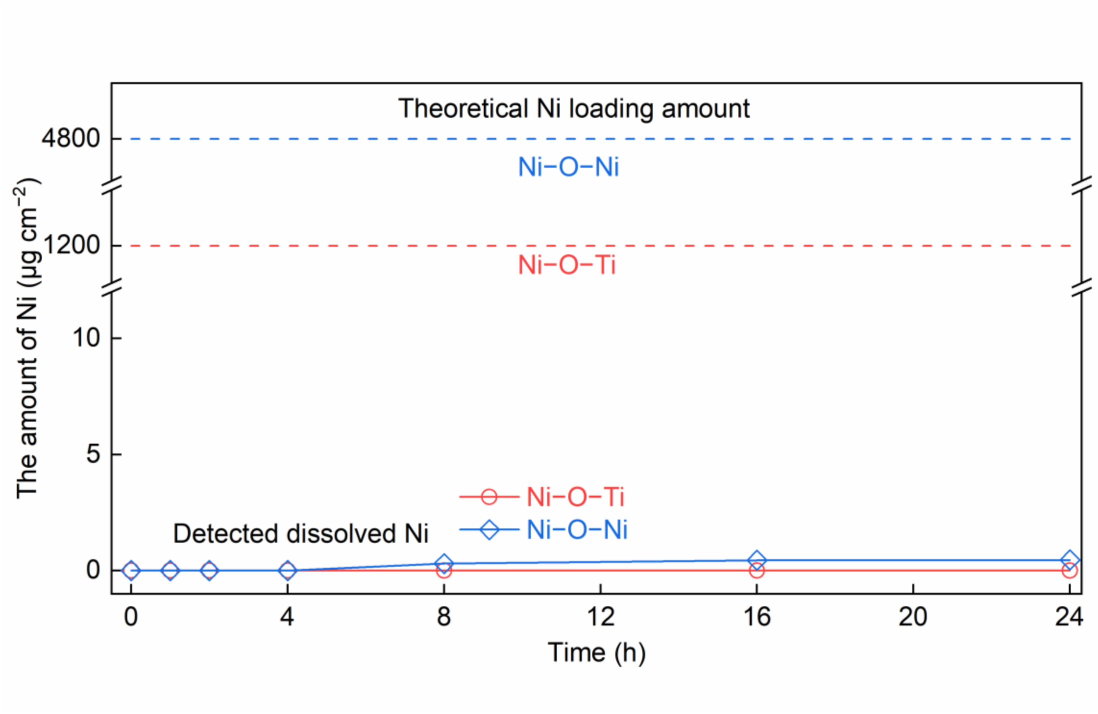

Supplementary Figure 29. The amount of Ni dissolved from asymmetric Ni–O–Ti and symmetric Ni–O–Ni sites during 24 hours durability test with the initial current density of 100 mA cm^{-2} in 1.0 M KOH containing 1.0 M urea.

The amount of potential dissolved Ni in aqueous solution was detected with Thermo Scientific iCAP^{TM} Q ICP-MS. It was found that the Ni dissolution from asymmetric Ni–O–Ti and symmetric Ni–O–Ni was less than 1.0 μg cm^{-2} after 24 hours of reaction, which was much lower than the theoretical Ni loading amount of Ni–O–Ti (1200 μg cm^{-2}) and Ni–O–Ni (4800 μg cm^{-2}) and ruled out the potential dissolution of Ni from the Ti foam.

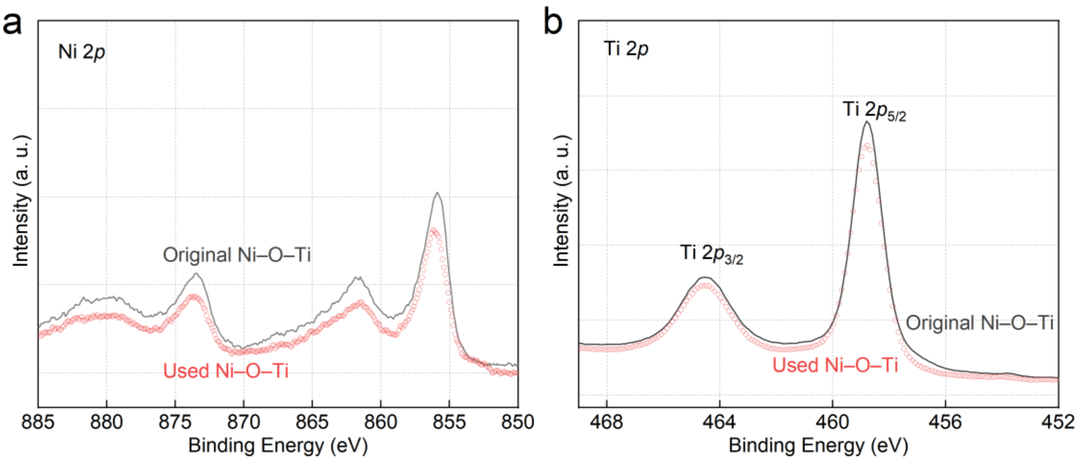

Supplementary Figure 30. High-resolution (a) Ni 2*p* and (b) Ti 2*p* XPS spectrum of original and long-term used Ti foam with atomically isolated asymmetric Ni–O–Ti sites.

We compared the high-resolution Ni 2*p* and Ti 2*p* XPS spectra of original and long-term used Ti foam loaded with Ni single atoms, and did not observe any obvious change of Ni 2*p* and Ti 2*p* XPS spectra after long-term electrochemical reaction. These results confirmed the excellent stability of Ni single atoms and Ti substrate during the reaction.

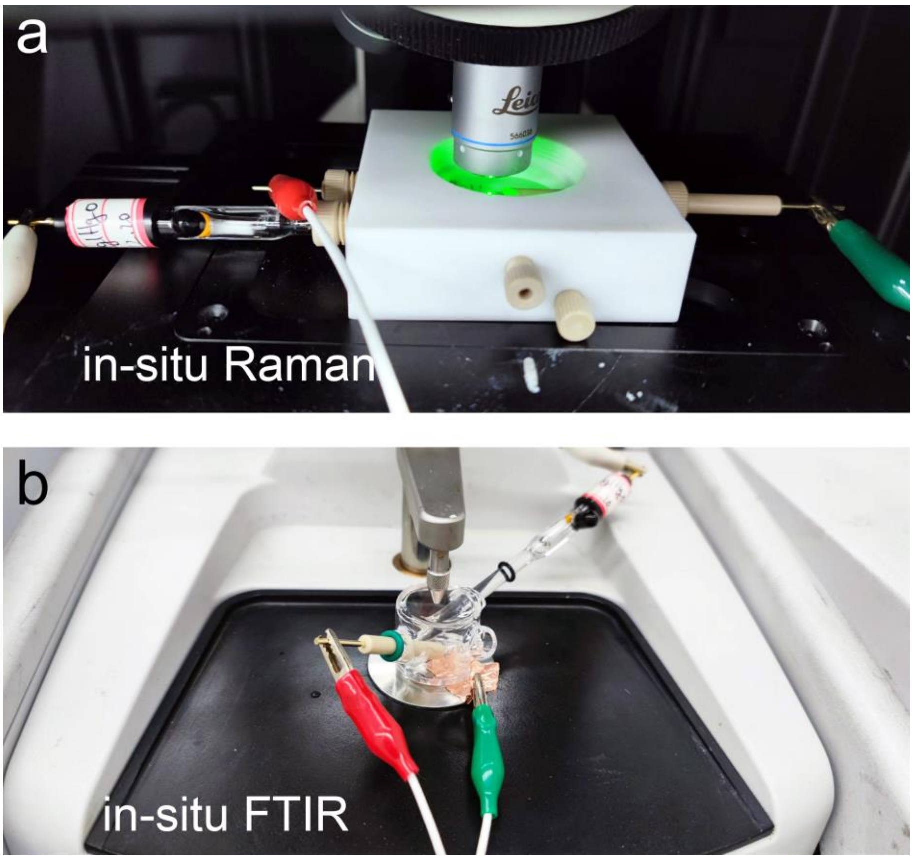

Supplementary Figure 31. The digital image of custom-built electrochemical cells for (a) in-situ Raman and (b) in-situ FTIR.

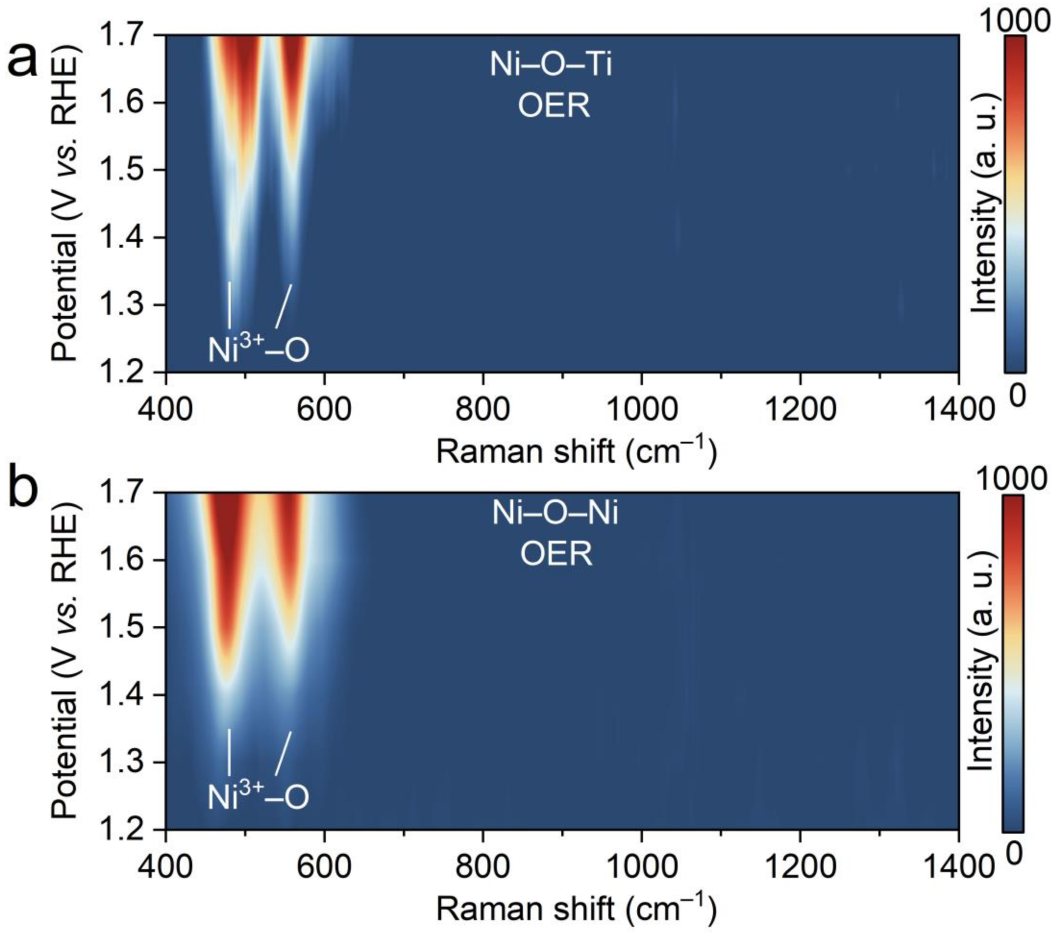

Supplementary Figure 32. In-situ Raman spectra of (a) atomically isolated Ni–O–Ti sites and (b) connected symmetric Ni–O–Ni sites were collected at different potentials during the OER operations in 1.0 M KOH. The signals appeared at 484 and 562 cm^{-1} was attributed to high-valent Ni^{3+}–O.

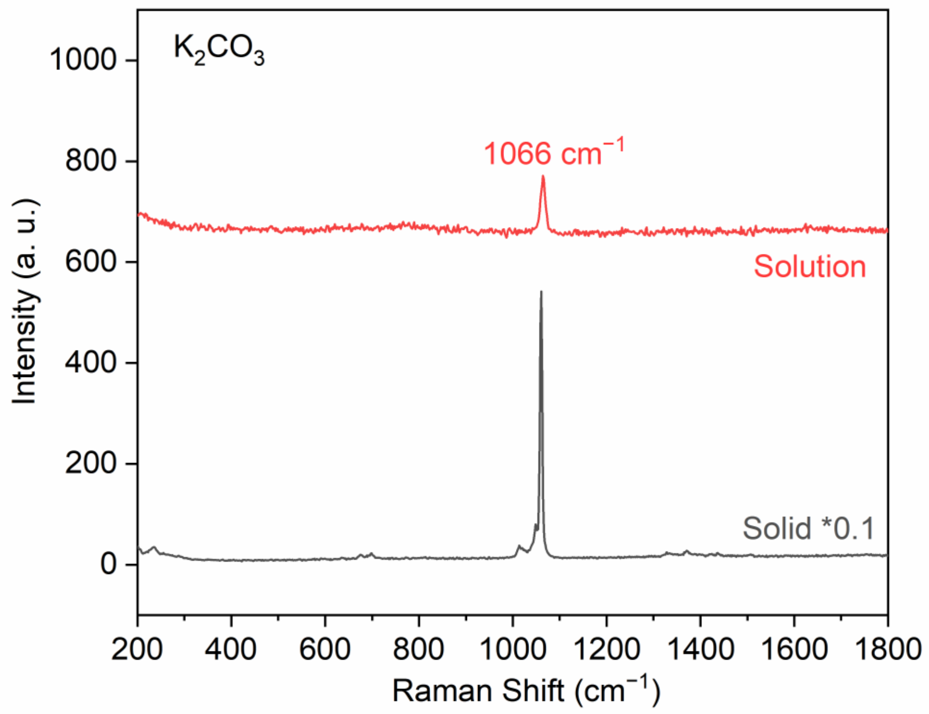

Supplementary Figure 33. The Raman spectra of solid and solution K_2CO_3 to identify the CO_3^{2-} species.

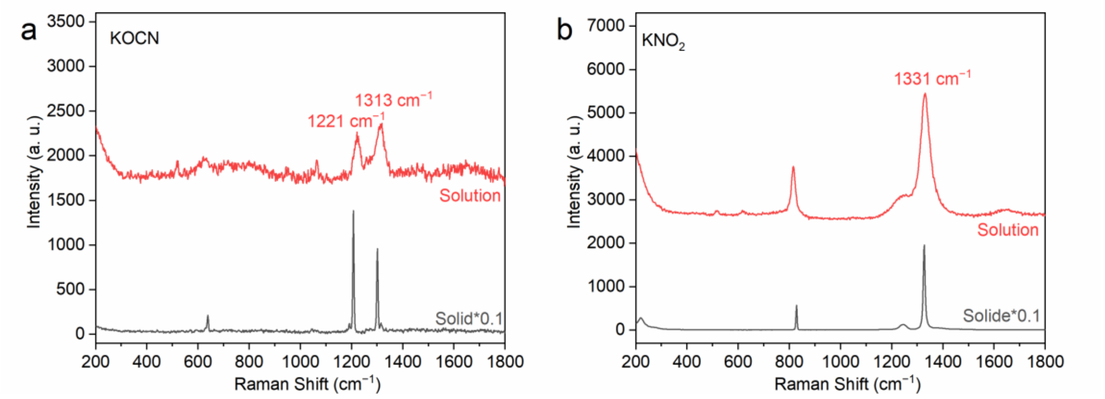

Supplementary Figure 34. The Raman spectra of solid and solution (a) KOCN and (b) KNO_2 to identify the NCO^- and NO_2^- species, respectively.

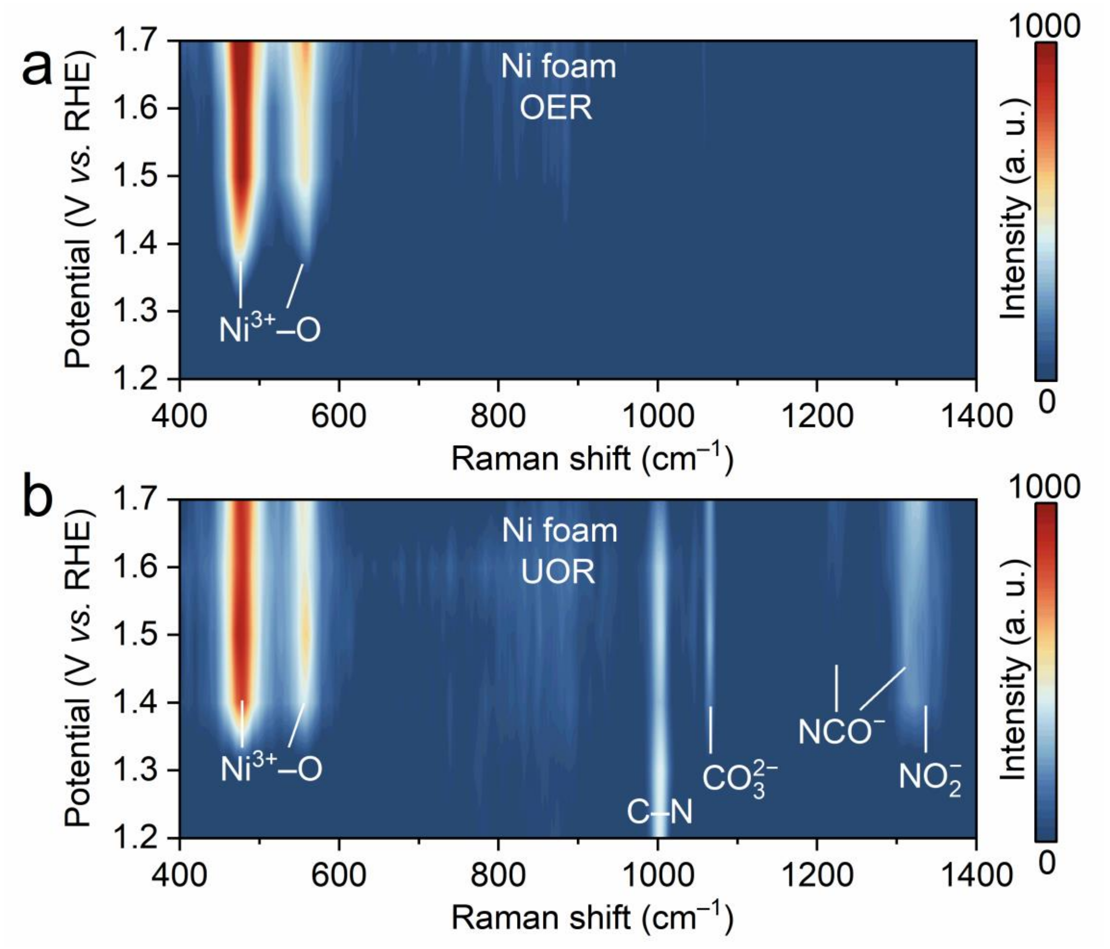

Supplementary Figure 35. In-situ Raman spectra of Ni foam were collected at different potentials during the (a) OER and (b) UOR operations. Due to the large number of Ni^{3+}–O species and weak UOR activity of Ni foam under applied potentials, the Ni^{3+}–O is not completely consumed and therefore is always present at 484 and 562 cm^{-1} during the UOR process. In contrast, the signals of CO_3^{2-} (1065 cm^{-1}), NCO$^-$ (1221 and 1313 cm^{-1}) and NO_2^- (1331 cm^{-1}) appeared gradually, indicating unsatisfactory UOR selectivity of Ni foam.

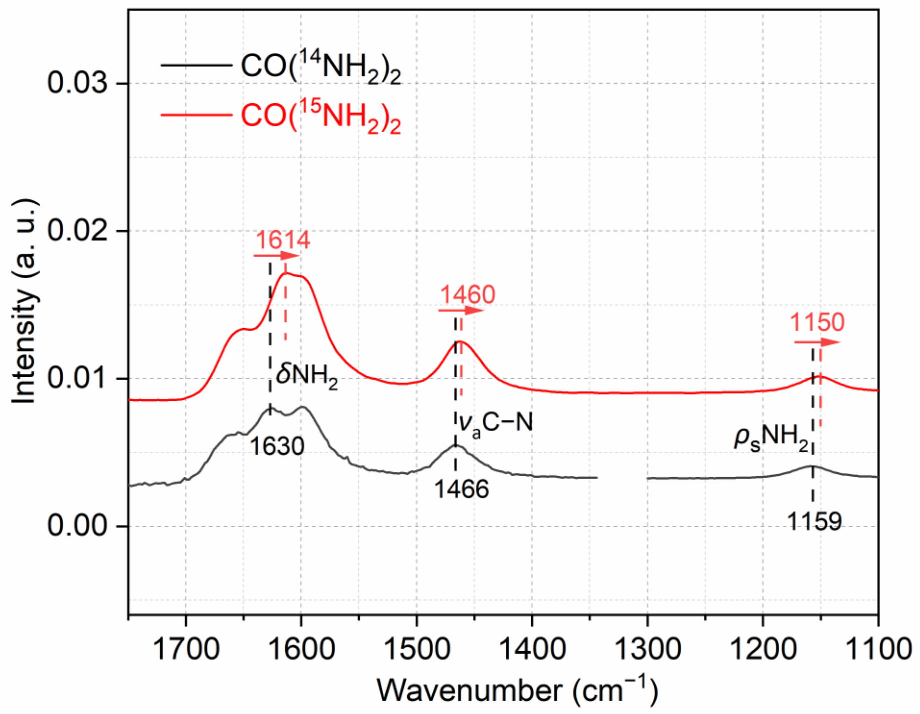

Supplementary Figure 36. The FTIR spectra of urea solution and adsorbed urea on atomically isolated asymmetric Ni–O–Ti sites and connected symmetric Ni–O–Ni sites.

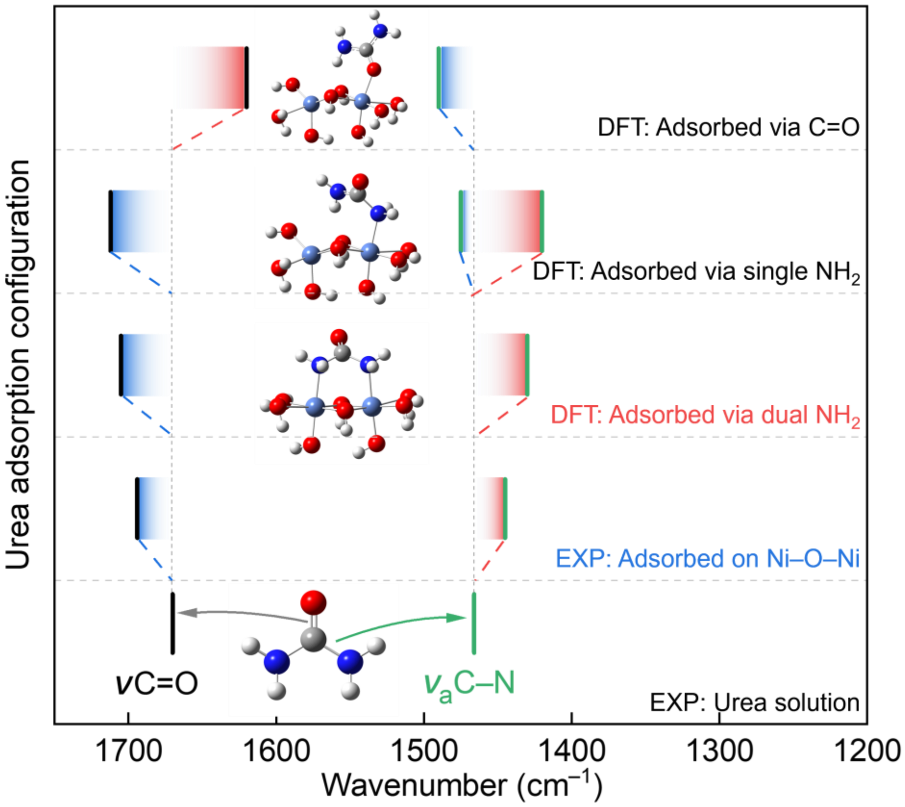

Supplementary Figure 37. The experimental FTIR frequencies of C=O and C⎓N of urea adsorbed at the Ni–O–Ni sites and theoretical frequencies of C=O and C⎓N of urea adsorbed at the Ni–O–Ni sites in different configurations calculated using Gaussian 09 package[3]. The computational models were optimized using open-shell UB3LYP method with the 6-31G* basis set[4]. SMD implicit solvent model were carried out to mimic the influence of water[5]. The frequencies of adsorbed urea were scaled by factor of 0.9614[6].

For the dual NH_2 adsorption model on Ni–O–Ni sites, our DFT calculations revealed a redshift in ν_a(C⎓N) due to the weakening of the C⎓N bond, and a blueshift in ν(C=O) resulting from the strengthening of the C=O bond. These computational findings were in alignment with the shifts we observed in the in-situ FTIR spectra and were consistent with previously reported data by Aldaz et al.[7] This model supported the dual NH_2 adsorption hypothesis at Ni–O–Ni sites. Conversely, for the terminal O adsorption on Ni–O–Ni sites, our simulations indicated a strengthening of the C⎓N bond and a weakening of the C=O bond, leading to a blueshift in ν_a(C⎓N) and a redshift in ν(C=O), respectively. These shifts differed from those observed at Ni–O–Ni sites and corroborated with experimental findings from Naguib et al.[8] These additional DFT results provided a solid scientific basis for our interpretations of the spectral changes observed in the in-situ FTIR studies.

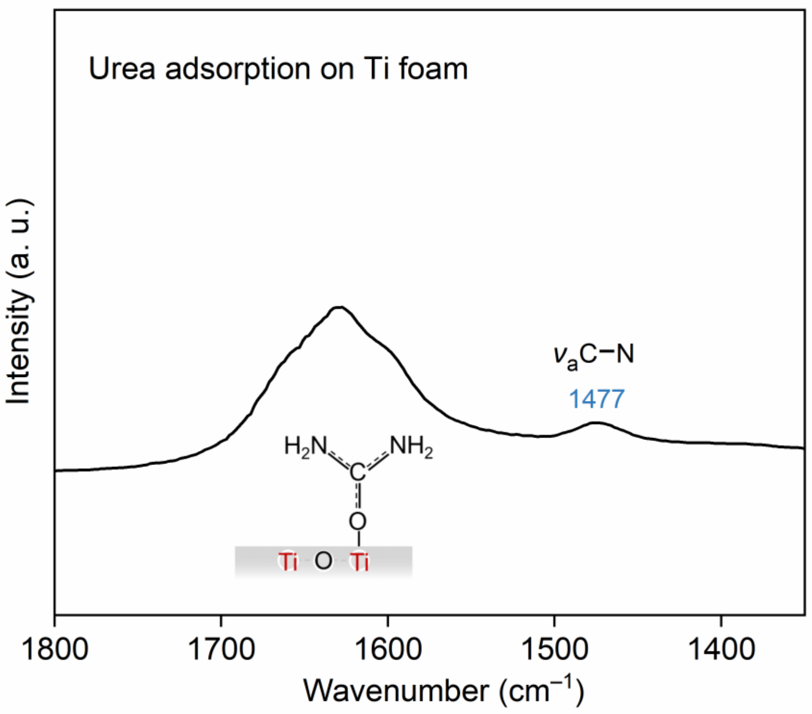

Supplementary Figure 38. The FTIR spectra of urea adsorbed on freestanding Ti foam. The inset shows the possible adsorption configuration of urea on Ti–O–Ti sites. The FTIR spectrum of urea on Ti foam was similar with the adsorption of urea on Ni–O–Ti sites, with a blueshift of v_a(C=N) frequency, suggesting that urea could be adsorbed on freestanding Ti foam.

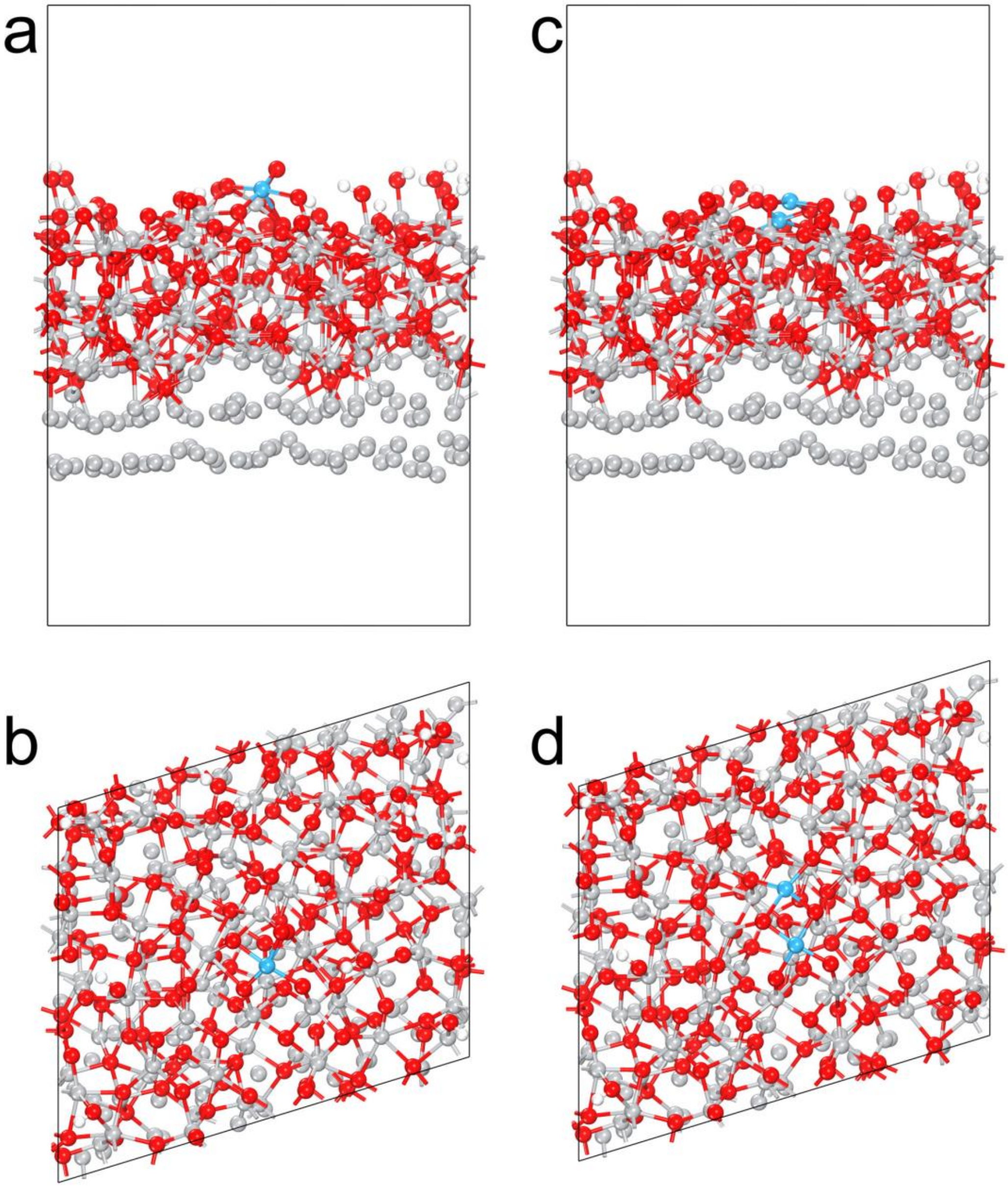

Supplementary Figure 39. Structural models of atomically isolated asymmetric Ni–O–Ti sites in (a) the side view and (b) the top view. Structural models of connected symmetric Ni–O–Ni sites in (c) the side view and (d) the top view. A slab including 2 atomic layers of Ti (001) with TiO_x surface obtained from ab-initio molecular dynamics (AIMD) in a 20.44 Å × 17.81 Å × 28.70 Å supercell are chosen to model the Ti foam with amorphous TiO_x (Supplementary Notes 4). Isolated Ni atom was constructed to simulate atomically isolated asymmetric Ni–O–Ti sites. Two adjacent Ni atoms were used to mimic the connected symmetric Ni–O–Ni sites.

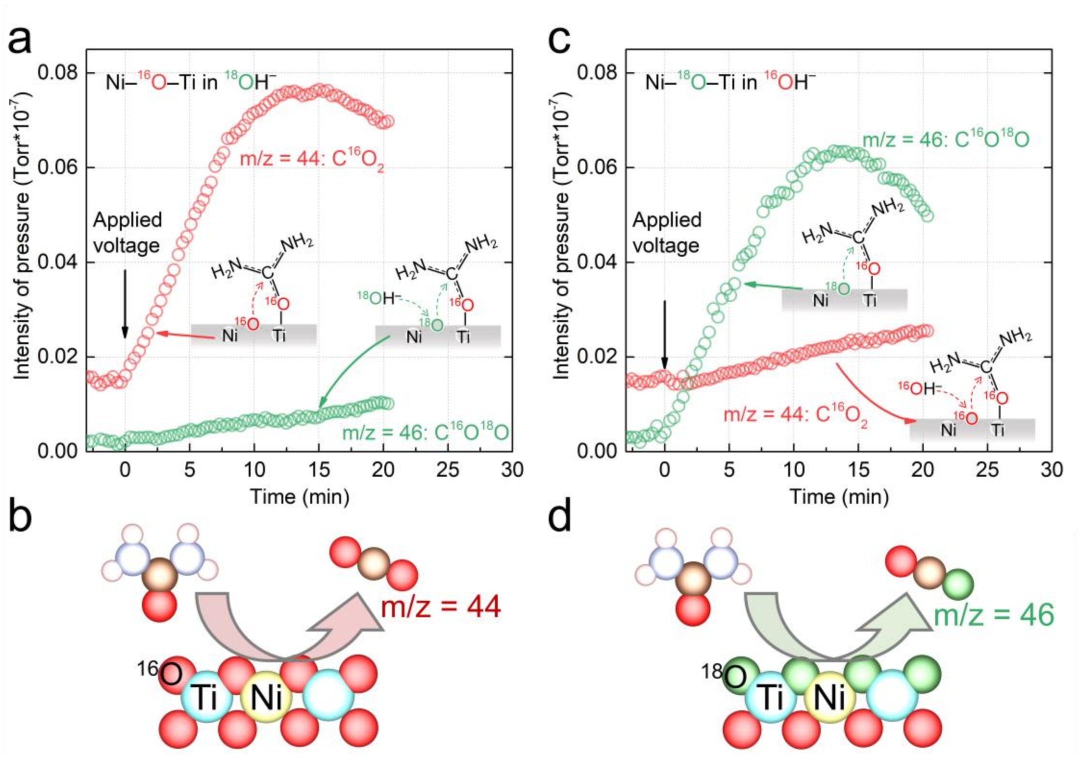

Supplementary Figure 40. (a) The on-line MS signals for m/z = 44 and 46 from anode chamber during UOR on Ni–^{16}O–Ti sites with the initial current density of 100 mA cm^{-2} by feeding 1.0 M K^{18}OH + 0.33 M urea (C^{16}O(NH$_2$)$_2$) in H$_2$^{18}O, and (b) corresponding schematic diagram of the on-line MS measurement process. (c) The on-line MS signals for m/z = 44 and 46 on ^{18}O labelled Ni–^{18}O–Ti sites during UOR by feeding 1.0 M K^{16}OH + 0.33 M urea (C^{16}O(NH$_2$)$_2$) in H$_2$^{16}O, and (d) corresponding schematic diagram of the on-line MS measurement process.

We employed on-line MS to check the participation of lattice O in the CO_2 generation during UOR in H$_2$^{18}O[9,10]. Initially, we observed the MS signal of C^{16}O$_2$ (m/z = 44) by feeding 1.0 M K^{18}OH + 0.33 M C^{16}O(NH$_2$)$_2$ in H$_2$^{18}O (Supplementary Fig. 40a and 40b), suggesting the participation of lattice ^{16}O from Ni–^{16}O–Ti sites. As the UOR progressed, the signals of C^{16}O^{18}O (m/z = 46) gradually increased, indicating that lattice ^{16}O atoms were gradually depleted and substituted by ^{18}O from the H$_2$^{18}O. After the lattice ^{16}O atoms in Ni–^{16}O–Ti were sufficiently replaced with ^{18}O atoms, the resulting Ni–^{18}O–Ti electrode was used for the electrooxidation of urea in H$_2$^{16}O containing 1.0 M K^{16}OH + 0.33 M C^{16}O(NH$_2$)$_2$. The instant production of C^{16}O^{18}O during the UOR strongly validated the direct involvement of lattice ^{18}O (Supplementary Fig. 40c and d). The lattice ^{18}O atoms in Ni–^{18}O–Ti were gradually substituted by ^{16}O from solution, leading to the production of C^{16}O$_2$.

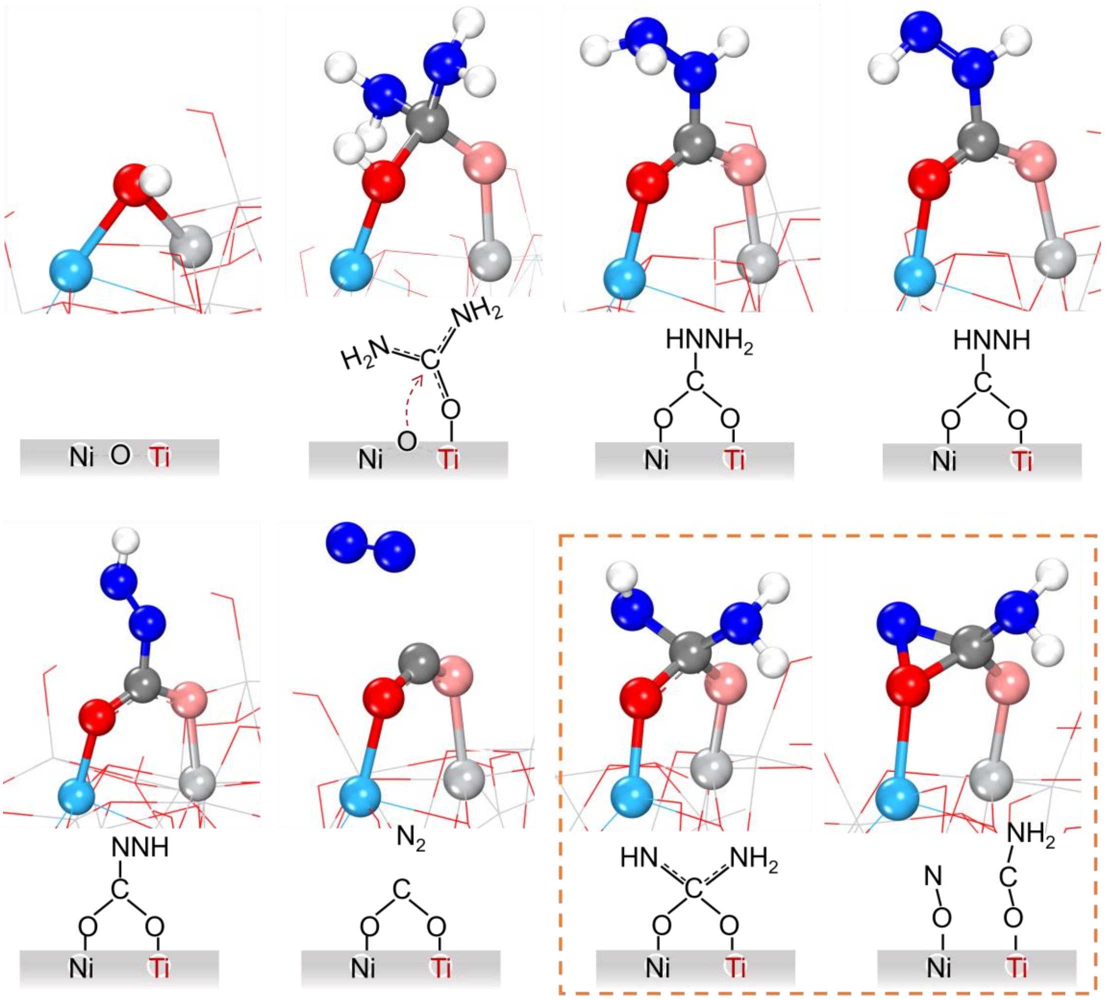

Supplementary Figure 41. The optimized structures via intermolecular N–N coupling and C=N cleavage (yellow dotted box) during UOR on atomically isolated asymmetric Ni–O–Ti sites.

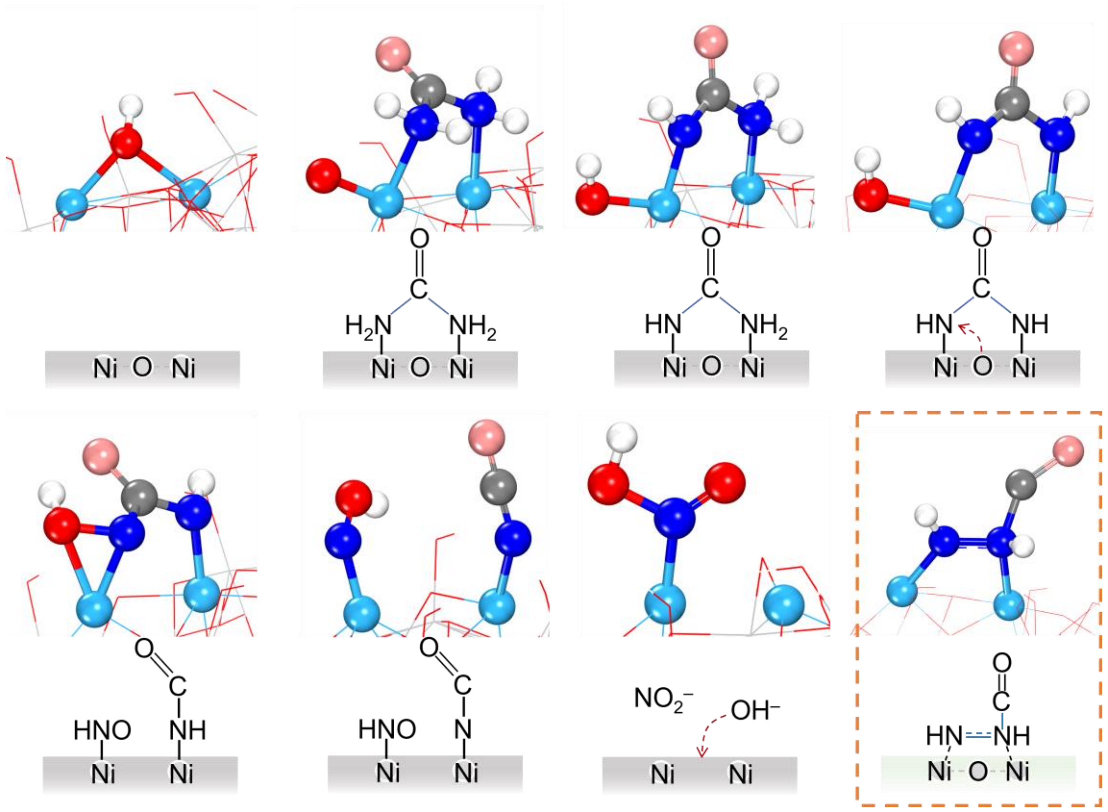

Supplementary Figure 42. The optimized structures via C⎓N cleavage and intermolecular N–N coupling (yellow dotted box) during UOR on connected symmetric Ni–O–Ni sites.

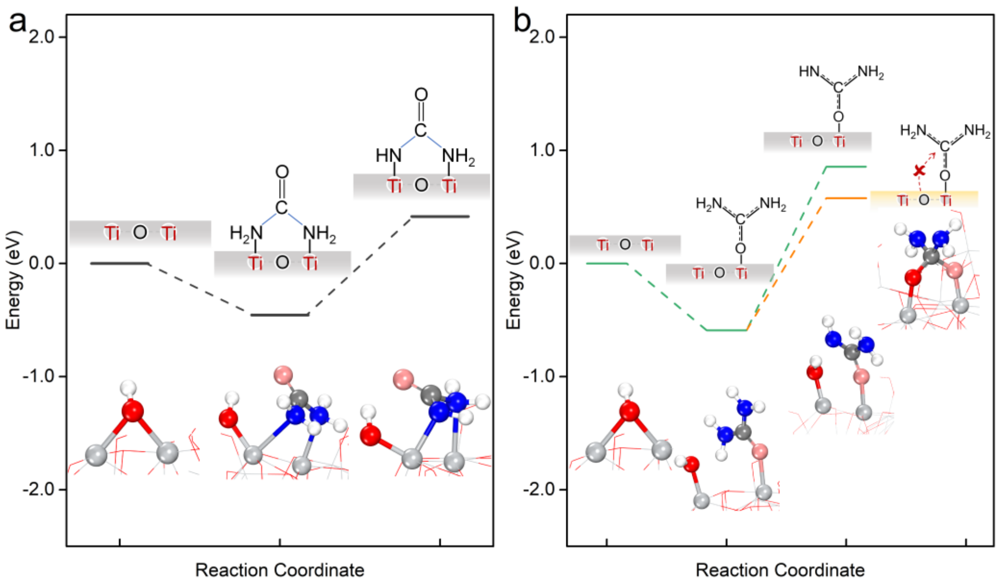

Supplementary Figure 43. The Gibbs free energy change and optimized structures of urea adsorption and the first deprotonation step at the symmetric Ti–O–Ti sites of freestanding Ti foam via interactions with (a) the C=O group and (b) NH_2 groups.

We performed DFT simulations to investigate the urea adsorption and the deprotonation step on symmetric Ti–O–Ti sites. Compared to urea adsorption via NH_2 groups on Ni–O–Ni sites (–0.46 eV, Supplementary Fig. 43a), urea prefers to the adsorption configuration on Ti–O–Ti sites via C=O group (–0.59 eV, Supplementary Fig. 43b), which is attributed to the high oxygenophilic affinity of Ti. However, the formation of sp^3-hybridized intermediates on Ti–O–Ti, through bounding with the C moiety of the C=O group similar with the case of Ni–O–Ti, is very difficult due to the required energy input of 0.84 eV. Without the introduction of Ni single atoms to generate high valent Ni(III), the energy requirement for the initial dehydrogenation step of urea is extremely high, no matter urea adsorption at Ti–O–Ti sites via NH_2 groups (0.86 eV) or C=O groups (1.45 eV). Therefore, the symmetric Ti–O–Ti sites were inert for UOR.

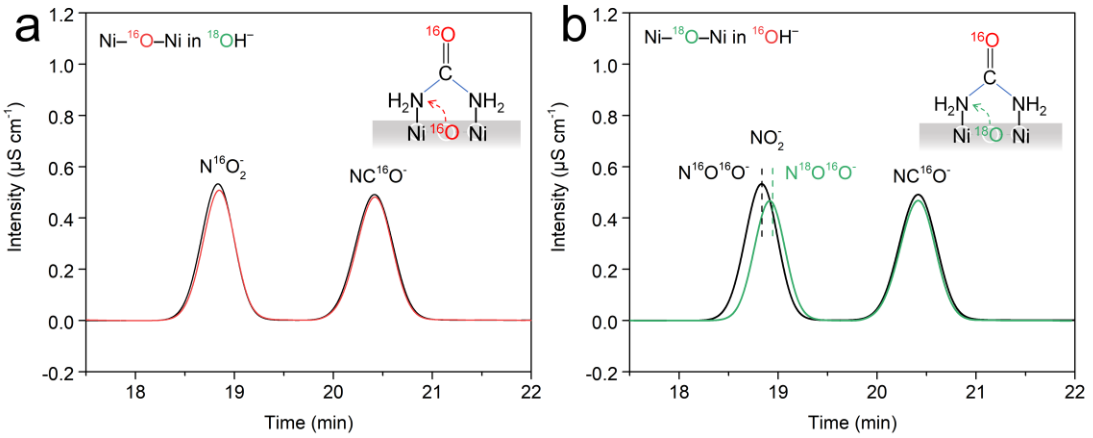

Supplementary Figure 44. The original data of ion chromatograms after the initial 10 min of UOR for connected symmetric (a) Ni–^{16}O–Ni sites by feeding 1.0 M K^{18}OH + 0.33 M urea in $H_2{}^{18}O$ solution and (b) ^{18}O labelled Ni–^{18}O–Ni sites by feeding 1.0 M K^{16}OH + 0.33 M urea in $H_2{}^{16}O$.

Ion chromatograms were used to check the participation of lattice O in the production of NO_2^- during UOR in $H_2{}^{18}O$. Only $N^{16}O_2^-$ was detected during the early stages of UOR of Ni–^{16}O–Ni sites by feeding 1.0 M K^{18}OH + 0.33 M urea ($C^{16}O(NH_2)_2$) in $H_2{}^{18}O$ solution (Supplementary Fig. 44a). For the Ni–^{18}O–Ni electrode, $N^{18}O^{16}O^-$ was generated during the early stages of UOR by feeding 1.0 M K^{16}OH + 0.33 M urea ($C^{16}O(NH_2)_2$) in $H_2{}^{16}O$ (Supplementary Fig. 44b). These experimental findings, supported by our DFT simulations, clearly demonstrated that lattice oxygen participates actively in the UOR process.

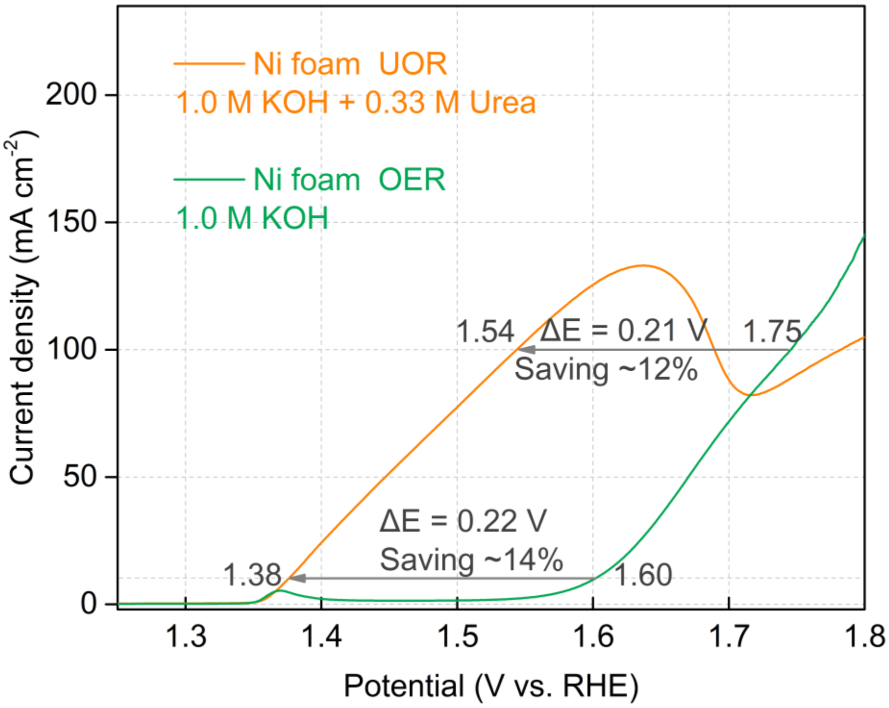

Supplementary Figure 45. LSV curves of Ni foam in 1.0 M KOH + 0.33 M urea and 1.0 M KOH for UOR and OER, respectively. The peak at 1.30–1.40 V_{RHE} corresponding to Ni^{2+} to Ni^{3+} transition with an onset potential of 1.30 V_{RHE}. Compared with OER, the potential reaching the current density of 100 mA cm^{-2} on Ni foam for UOR was reduced by 0.21 V, confirming that UOR was more thermodynamically favorable than OER and could save ~12% energy for hydrogen generation.

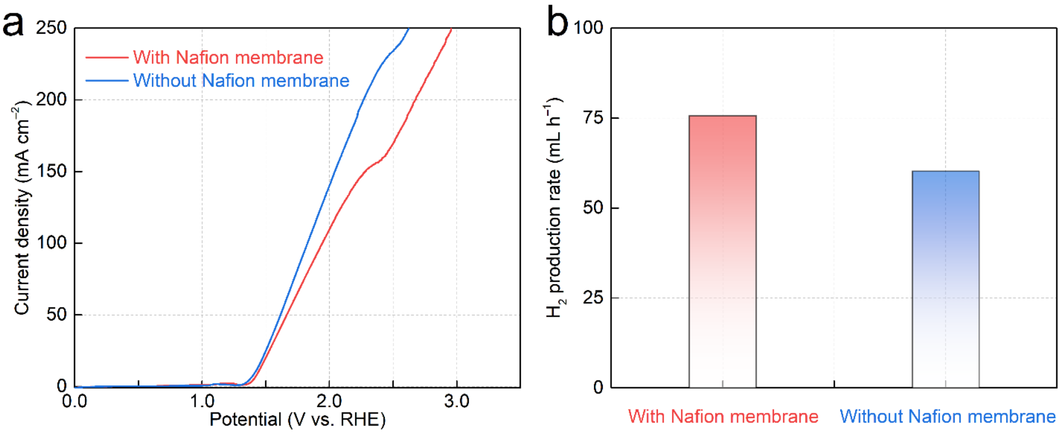

Supplementary Figure 46. (a) The UOR polarization curves with and without Nafion membrane using asymmetric Ni–O–Ti sites as the anode and Pt foil as the cathode, and (b) corresponding hydrogen production rate.

Although the Nafion membrane increased the resistance and reduced the current density of the system (Supplementary Fig. 46a), it separated the generated H_2 and avoided its oxidation at the anode, thus improving the efficiency of hydrogen production (Supplementary Fig. 46b).

Supplementary Notes 1.

The computation of annually urea production derived from human urine considers a global population of an estimated 8.0 billion people, each producing an average of 20 grams of urea daily. This total is further multiplied by 365 days to encapsulate the annually urea production[11,12].

$$20\ \text{g} * 8.0 * 10^9 * 365 = 58.4\ \text{Megatons}$$

Supplementary Notes 2.

The energy-saving efficiency (η) in H_2 production was calculated according to the following equation:

$$\eta = (E_{OER} - E_{UOR})/E_{OER}$$

E_{OER} and E_{UOR} represent the potentials that reach a specific current density for OER and UOR, respectively. For a current density of 100 mA cm^{-2} for Ni–O–Ti, the potentials required for OER and UOR were 1.61 and 1.33 V_{RHE}, respectively. Therefore, the energy-saving efficiency in H_2 production by Ni–O–Ti was 17% according to the above equation.

Supplementary Notes 3.

Synthesis of Ni_1@NC. In a typical procedure, 0.546 g of $Ni(NO_3)_2 \cdot 6H_2O$ was first dissolved in 15.0 mL methanol to form a homogeneous solution, which was subsequently injected into 15.0 mL of methanol containing 0.616 g 2-methylimidazole under ultrasound for 10 min at room temperature. The mixed solution was then transferred into 50 mL Teflon-lined stainless-steel autoclaves and heated at 120 ℃ for 4 h to obtain ZIF-67. Finally, the ZIF-67 were washed with ethanol for several times and further dried in vacuum at 70℃ for post-treatment. The as-prepared ZIF-67 powder was placed in a tube furnace and then heated to 800 ℃ for 3 h at a heating rate of 5 ℃/min under N_2 atmosphere, and then naturally cooled to room temperature to obtain the Ni_1@NC. Inductively coupled plasma-mass spectrometry (ICP-MS) showed a cobalt loading of 1.46 wt% on Ni_1@NC.

Supplementary Notes 4.

XAFS measurements. The X-ray absorption fine structure spectra of Ni K-edge was obtained at Singapore Synchrotron Light Source center (SSLS, operating at 2.5 GeV with a maximum current of 200 mA). The EXAFS spectra were determined via subtracting the post-edge background from the overall absorption and then normalized to the edge-jump step. Subsequently, the $\chi(\mathbf{k})$ data was Fourier transformed to real (R) space by using a hanning windows ($d\mathbf{k}$ =1.0 Å^{-1}) to separate the EXAFS contributions from different coordination shells. To obtain the quantitative structural parameters around central atoms, least-squares curve parameter fitting was performed with using the ARTEMIS module of IFEFFIT software packages.

Supplementary Notes 5.

DFT calculations. Spin-polarized DFT calculations were performed by the Vienna Ab initio Simulation Package The exchange-correlation interaction was described by the Perdew–Burke–Ernzerhof functional within the generalized gradient approximation (GGA)[13]. The core electrons were described by the projector augmented wave (PAW) pseudopotential wave. The effect of van der Waals interaction was described using the dispersion-corrected DFT-D3 functional[14]. The valence electrons included in the self-consistent-field calculations for each atom are listed: Ti_sv ($3s^2p^6d^2$ $4s^2$), Ni ($3d^8$ $4s^2$), O ($2s^2p^4$), N ($2s^2p^3$), C ($2s^2p^2$), and H ($1s^1$). DFT+U calculations were performed with U_{eff} values of 3.5 and 2 eV for Ti 3d and Ni 3d to correct the strong electron-correlation[15].

A representative two atomic layers of Ti metal covered with amorphous TiO_x loaded with an isolated Ni atom was constructed to simulate atomically isolated asymmetric Ni–O–Ti sites in a 20.44 Å × 17.81 Å × 28.70 Å supercell using ab-initio molecular dynamics (AIMD)[16–18]. Two adjacent Ni atoms were used to mimic the connected symmetric Ni–O–Ni sites. A vacuum space along the z direction was set to 15 Å to prevent the interaction between the repeating slabs. A k-point mesh of 1 × 1 × 1 was used to sample the slab Brillouin zone. A fixed volume canonical ensemble was applied to conduct anneal-to-quench process from 1500 K to 298 K with a series of AIMD simulations, and each experiment was conducted at a constant temperature using Nose–Hoover method[19]. Initial temperature of 1500 K was chosen to melt the

TiO_x surface layer of the Ti foam, and 3.0 ps with a time step of 1.0 fs was given to optimize the configuration. The structure obtained from previous AIMD simulation at 1500 K was used as the input structure for 298 K, and ran for 10 ps to fully reach its equilibrium configuration using the gamma point of the Brillouin zone. The cutoff energy of the plane-wave basis was 400 eV, and the convergence accuracy of total energy for each step was less than 1×10^{-5} eV per atom in the AIMD simulation. The obtained single-atom Ni coordinated with five O atoms forming five-coordinate configuration, in line well with the EXAFS result.

During the geometrical optimization, the kinetic cutoff energy was 420 eV with a force convergence of 0.02 eV/Å. Thermal and zero-point energy (ZPE) corrections were calculated over Γ points. The Gaussian smearing method with an electronic temperature of $k_BT = 0.05$ eV was employed. The calculation of the free energy diagrams was performed by using the concept of computational hydrogen electrode (CHE)[20]. The adsorption energies (E_{ads}) of the adsorbed species were described by the following equation:

$$E_{ads} = E_{total} - E_{adsorbate} - E_{slab}$$

where E_{total}, $E_{adsorbate}$ and $E_{surface}$ are the total energies of the electrocatalyst–adsorbate complex, the adsorbate and electrocatalyst, respectively. The Gibbs free energy change (ΔG) of the elementary step was estimated using:

$$\Delta G = \Delta E_{DFT} + \Delta E_{ZPE} - T\Delta S$$

where ΔE_{DFT} is the change in the electronic energy difference calculated by DFT, ΔE_{ZPE} is the change in the zero–point energy, T is the temperature (298.15 K) and ΔS is the entropy change. The ZPE and ΔS of the UOR species were computed from the vibrational frequencies over G points, in which only the adsorbate vibrational modes were calculated explicitly, while the electrocatalyst was fixed. The crystal orbital Hamilton population (COHP) was analyzed by the LOBSTER package with a denser k-point mesh of 3 × 3 × 1[21–24].

Supplementary Notes 6.

The RHE calibration of the Hg/HgO reference electrode was performed in a high purity H_2-saturated 1.0 M KOH with a platinum RDE (PINE, 0.126 cm^2) as the working electrode, Pt foil as the counter electrode, and Hg/HgO (1.0 M KOH) as the reference electrode. CV measurements was conducted at a scan rate of 1 mV s^{-1}, and the average of the two potentials at which the current crossed zero was taken as the thermodynamic potential for the hydrogen electrode reaction. In 1.0 M KOH solution, the RHE was calibrated as the follow equation: $E_{RHE} = E_{Hg/HgO} + 0.908$ V.

Supplementary Table 1. The theoretical loading and actual loading weight percentages (wt.%) according to ICP-MS.

Samples	Ni^{2+} solution (μL)	Theoretical	ICP-MS
0.06%Ni–O–Ti	50.0	0.06 wt.%	0.07 wt.%
0.15%Ni–O–Ti	100.0	0.15 wt.%	0.15 wt.%
0.3%Ni–O–Ti	200.0	0.30 wt.%	0.29 wt.%
0.3%Ni–O–Ti after long-term stability test	/	/	0.28 wt.%
1.2%Ni–O–Ni	800.0	1.20 wt.%	1.08 wt.%
Ni_1@NC	/	/	1.46 wt.%

The theoretical loading (η) was calculated based on the following equation:

$$\eta = V*c/m \times 100\%$$

where V refers to the volume of $NiCl_2 \cdot 6H_2O$ ethanol solution utilized during the preparation, c refers to the Ni mass concentration of the $NiCl_2 \cdot 6H_2O$ ethanol solution (3.0 mg mL^{-1}), and m refers to the mass of Ti foam electrode. To illustrate, consider 0.3%Ni–O–Ti. The mass of Ti foam electrode measuring 1.0 cm × 0.5 cm × 0.68 mm was approximately 200 mg, and the corresponding V was reported to be 200.0 μL. Following the given formula, the theoretical capacity loading was calculated as 0.3%.

The amount of Ni on Ti foam remained virtually unaltered after long-term testing, thereby validating the thermodynamic stability of the Ni–O–Ti sites.

Supplementary Table 2. The fitting parameters of Ni K-edge Fourier-filtered **k**^{3}-weighted EXAFS for various samples.

Samples	Path	CN[a]	R (Å)[b]	σ^2 (10^{-3}Å^2)[c]	ΔE_0 (eV)	R_f (%)
Ni foil	Ni–Ni	12	2.48 ± 0.01	5.9 ± 0.5	–5.4 ± 0.8	0.53
NiO	Ni–O	6	2.08 ± 0.01	6.7 ± 0.8	–1.0 ± 0.3	0.44
	Ni–Ni	12	2.95 ± 0.01	6.6 ± 0.6	–3.2 ± 0.4	
$Ni(OH)_2$	Ni–O	6	2.05 ± 0.01	6.6 ± 0.7	–3.9 ± 0.7	0.22
	Ni–Ni	6	3.12 ± 0.01	6.9 ± 0.4	–1.1 ± 0.8	
Asymmetric Ni–O–Ti	Ni–O	4.5	2.03 ± 0.01	3.5 ± 0.2	4.9 ± 0.9	0.52
	Ni–Ti	4.8	2.99 ± 0.02	9.2 ± 0.4	8.0 ± 0.8	
Symmetric Ni–O–Ti	Ni–O	1.1	2.02 ± 0.02	8.5 ± 0.8	6.5 ± 0.7	0.67
	Ni–Ni	4.1	2.50 ± 0.03	9.5 ± 0.5	–6.1 ± 0.4	
	Ni–Ti	1.5	2.96 ± 0.05	12.1 ± 0.7	8.2 ± 0.5	

[a]CN: coordination numbers; [b]R: the interatomic distance (the bond length between central atoms and surrounding coordination atoms); [c]σ^2: Debye–Waller factor (a measure of thermal and static disorder in absorber-scatterer distances); ΔE_0: edge-energy shift (the difference between the zero kinetic energy value of the sample and that of the theoretical model). R factor: goodness of fit. S_0^2 was set to 0.77 for Ni foil according to the experimental EXAFS fit of Ni foil by fixing CN as the known crystallographic value. For Ni foil EXAFS fitting, the data ranges are presented as follows: $3.0 \le k \le 12.0$ Å^{-1}, $1.0 \le R \le 3.0$ Å. The independent point is 11.5 and the number of variables is 4. For NiO EXAFS fitting, the S_0^2 was set to 0.90. The data ranges are presented as follows: $3.0 \le k \le 12.0$ Å^{-1}, $1.0 \le R \le 3.0$ Å. The independent point is 11.4 and the number of variables is 7. For $Ni(OH)_2$ EXAFS fitting, the S_0^2 was set to 0.97. The data ranges are presented as follows: $3.0 \le k \le 11.0$ Å^{-1}, $1.0 \le R \le 3.4$ Å. The independent point is 12.2 and the number of variables is 7. For asymmetric Ni–O–Ti EXAFS fitting, the data ranges are presented as follows: $3.0 \le k \le 11.0$ Å^{-1}, $1.0 \le R \le 3.5$ Å. The independent point is 12.7 and the number of variables is 11. For symmetric Ni–O–Ni EXAFS fitting, the data ranges are presented as follows: $3.0 \le k \le 11.5$ Å^{-1}, $1.0 \le R \le 3.5$ Å. The independent point is 13.5 and the number of variables is 12.

Supplementary Table 3. The comparison of UOR performances of the catalysts in this work and other reported Ni-based catalysts.

Catalyst	Potential (V) (10 mA cm^{-2})	Potential (V) (100 mA cm^{-2})	Reference
Ni–O–Ti	1.30	1.33	This Work
Ni–O–Ni	1.31	1.34	
Ni foam	1.38	1.54	
Ni_1@NC	1.38	/	
Mn-Ni_3S_2/NF-0.2	1.303	1.397	Ref [25]
P-$CoNi_2S_4$	1.306	1.367	Ref [26]
β-$Co_{0.1}Ni_{0.9}(OH)_2$/NF	1.31	1.34	Ref [27]
$Ni_2Fe(CN)_6$	1.33	1.35	Ref [28]
Ni(OH)S/NF	1.34	1.37	Ref [29]
O-NiMoP/NF	1.35	1.41	Ref [30]
Fe-doped NiS-NiS_2	1.35	1.37	Ref [31]
NiOOH/(LDH/α-FeOOH)	1.35	1.40	Ref [32]
Co,V co-doped NiS_2	1.35	1.55	Ref [33]
Ni-Mo nanotubes	1.36	1.43	Ref [34]
Ni/FeOOH	1.373	1.407	Ref [35]
Ni-S-Se/NF	1.38	1.42	Ref [36]

Supplementary Table 4. The comparison of UOR selectivity to N_2 of the catalysts in this work and other reported Ni-based catalysts.

Electrocatalyst	selectivity of N_2	Electrolyte	Reference
Ni–O–Ti	99%	1.0 M KOH + 0.33 M urea 1.40 V vs. RHE	This Work
Ni–O–Ni	60%		
Ni foam	23%		
Ni_1@NC	9.9%		
Cu/Ni-B	70%	1.0 M KOH + 0.33 M urea	Ref [37]
$Ni_{0.8}Cu_{0.2}(OH)_2$	55%	1.0 M KOH + 0.33 M urea	Ref [38]
$Ni(OH)_2$	30%	1.0 M KOH + 0.33 M urea	
PANI-Ac-NF	31.1%	1.0 M KOH + 0.33 M urea	Ref [39]
Ac-NF	15%	1.0 M KOH + 0.33 M urea	
$NiCo_2O_4$ nanosheets	89%	5.0 M KOH + 0.33 M urea	Ref [40]

Supplementary References

1. Yao, Y., et al. Single Atom Ru Monolithic Electrode for Efficient Chlorine Evolution and Nitrate Reduction. *Angew. Chem. Int. Ed.* **61**, e202208215 (2022).
2. Cai, M., et al. Formation and Stabilization of NiOOH by Introducing α-FeOOH in LDH: Composite Electrocatalyst for Oxygen Evolution and Urea Oxidation Reactions. *Adv. Mater.* **35**, 2209338 (2023).
3. Frisch, M. J., et al. Gaussian 09 Rev. A. 1, (2009).
4. Stephens, P. J., Devlin, F. J., Chabalowski, C. F. & Frisch, M. J. Ab Initio Calculation of Vibrational Absorption and Circular Dichroism Spectra Using Density Functional Force Fields. *J. Phys. Chem.* **98**, 11623–11627 (1994).
5. Marenich, A. V., Cramer, C. J. & Truhlar, D. G. Universal Solvation Model Based on Solute Electron Density and on a Continuum Model of the Solvent Defined by the Bulk Dielectric Constant and Atomic Surface Tensions. *J. Phys. Chem. B* **113**, 6378–6396 (2009).
6. Scott, A. P. & Radom, L. Harmonic Vibrational Frequencies: An Evaluation of Hartree−Fock, Møller−Plesset, Quadratic Configuration Interaction, Density Functional Theory, and Semiempirical Scale Factors. *J. Phys. Chem.* **100**, 16502–16513 (1996).
7. Climent, V., et al. On the Electrochemical and in-Situ Fourier Transform Infrared Spectroscopy Characterization of Urea Adlayers at Pt(100) Electrodes. *Langmuir* **13**, 2380–2389 (1997).

8. Overbury, S. H., et al. Complexity of Intercalation in MXenes: Destabilization of Urea by Two-Dimensional Titanium Carbide. *J. Am. Chem. Soc.* **140**, 10305–10314 (2018).

9. Ping, X., et al. Locking the lattice oxygen in RuO_2 to stabilize highly active Ru sites in acidic water oxidation. *Nat. Commun.* **15**, 2501 (2024).

10. Grimaud, A., et al. Activating lattice oxygen redox reactions in metal oxides to catalyse oxygen evolution. *Nat. Chem.* **9**, 457–465 (2017).

11. Boggs, B. K., King, R. L. & Botte, G. G. Urea electrolysis: direct hydrogen production from urine. *Chem. Commun.*, 4859–4861 (2009).

12. Rollinson, A. N., Jones, J., Dupont, V. & Twigg, M. V. Urea as a hydrogen carrier: a perspective on its potential for safe, sustainable and long-term energy supply. *Energy Environ. Sci.* **4**, 1216–1224 (2011).

13. Perdew, J. P., Burke, K. & Ernzerhof, M. Generalized Gradient Approximation Made Simple. *Phys. Rev. Lett.* **77**, 3865–3868 (1996).

14. Grimme, S., Antony, J., Ehrlich, S. & Krieg, H. A consistent and accurate ab initio parametrization of density functional dispersion correction (DFT-D) for the 94 elements H-Pu. *J. Chem. Phys.* **132**, 154104 (2010).

15. Dudarev, S. L., Botton, G. A., Savrasov, S. Y., Humphreys, C. J. & Sutton, A. P. Electron-energy-loss spectra and the structural stability of nickel oxide: An LSDA+U study. *Phys. Rev. B* **57**, 1505–1509 (1998).

16. Hu, Y., et al. Single Ru Atoms Stabilized by Hybrid Amorphous/Crystalline FeCoNi Layered Double Hydroxide for Ultraefficient Oxygen Evolution. *Adv. Energy Mater.* **11**, 2002816 (2021).

17. Wang, J., et al. Engineering the Coordination Environment of Ir Single Atoms with Surface Titanium Oxide Amorphization for Superior Chlorine Evolution Reaction. *J. Am. Chem. Soc.* **146**, 11152–11163 (2024).

18. Wang, J., et al. Amorphization activated ruthenium-tellurium nanorods for efficient water splitting. *Nat. Commun.* **10**, 5692 (2019).

19. Martyna, G. J., Klein, M. L. & Tuckerman, M. Nosé–Hoover chains: The canonical ensemble via continuous dynamics. *J. Chem. Phys.* **97**, 2635–2643 (1992).

20. Norskov, J. K., et al. Trends in the exchange current for hydrogen evolution. *J. Electrochem. Soc.* **152**, J23–J26 (2005).

21. Maintz, S., Deringer, V. L., Tchougreeff, A. L. & Dronskowski, R. LOBSTER: A tool to extract chemical bonding from plane-wave based DFT. *J. Comput. Chem.* **37**, 1030–1035 (2016).

22. Nelson, R., et al. LOBSTER: Local orbital projections, atomic charges, and chemical-bonding analysis from projector-augmented-wave-based density-functional theory. *J. Comput. Chem.* **41**, 1931–1940 (2020).

23. Dronskowski, R. & Blochl, P. E. Crystal orbital Hamilton populations (COHP): energy-resolved visualization of chemical bonding in solids based on density-functional calculations. *J. Phys. Chem.* **97**, 8617–8624 (1993).

24. Deringer, V. L., Tchougreeff, A. L. & Dronskowski, R. Crystal orbital Hamilton population (COHP) analysis as projected from plane-wave basis sets. *J. Phys. Chem. A* **115**, 5461–5466 (2011).

25. Yang, H., et al. In Situ Construction of a Mn^{2+}-Doped Ni_3S_2 Electrode with Highly Enhanced Urea Oxidation Reaction Performance. *ACS Sustain. Chem. Eng.* **8**, 8348–8355 (2020).

26. Lu, X. F., Zhang, S. L., Sim, W. L., Gao, S. & Lou, X. W. Phosphorized $CoNi_2S_4$ Yolk–Shell Spheres for Highly Efficient Hydrogen Production via Water and Urea Electrolysis. *Angew. Chem. Int. Ed.* **60**, 22885–22891 (2021).

27. Chen, W., et al. Unveiling the Electrooxidation of Urea: Intramolecular Coupling of the N−N Bond. *Angew. Chem. Int. Ed.* **60**, 7297–7307 (2021).

28. Geng, S.-K., et al. Nickel ferrocyanide as a high-performance urea oxidation electrocatalyst. *Nat. Energy* **6**, 904–912 (2021).

29. Jia, X., et al. Amorphous Ni(III)-based sulfides as bifunctional water and urea oxidation anode electrocatalysts for hydrogen generation from urea-containing water. *Appl. Catal. B* **312**, 121389 (2022).

30. Jiang, H., et al. Oxygen-Incorporated NiMoP Nanotube Arrays as Efficient Bifunctional Electrocatalysts For Urea-Assisted Energy-Saving Hydrogen Production in Alkaline Electrolyte. *Adv. Funct. Mater.* **31**, 2104951 (2021).

31. Huang, S., et al. Construction of Fe-doped NiS–NiS_2 Heterostructured Microspheres Via Etching Prussian Blue Analogues for Efficient Water-Urea Splitting. *Small* **18**, 2106841 (2022).

32. Cai, M., et al. Formation and Stabilization of NiOOH by Introducing α-FeOOH in LDH: Composite Electrocatalyst for Oxygen Evolution and Urea Oxidation Reactions. *Adv. Mater.* **35**, 2209338 (2022).

33. Ji, Z., et al. Pathway Manipulation via Ni, Co, and V Ternary Synergism to Realize High Efficiency for Urea Electrocatalytic Oxidation. *ACS Catal.* **12**, 569–579 (2021).

34. Zhang, J., et al. Energy-saving hydrogen production coupling urea oxidation over a bifunctional nickel-molybdenum nanotube array. *Nano Energy* **60**, 894–902 (2019).

35. Zhang, J., Bao, W., Li, M., Yang, C. & Zhang, N. Ultrafast formation of an FeOOH electrocatalyst on Ni for efficient alkaline water and urea oxidation. *Chem. Commun.* **56**, 14713–14716 (2020).

36. Chen, N., Du, Y., Zhang, G., Lu, W. & Cao, F. Amorphous nickel sulfoselenide for efficient electrochemical urea-assisted hydrogen production in alkaline media. *Nano Energy* **81**, 105605 (2021).

37. Shen, Z., Qi, Y., Ge, W., Jiang, H. & Li, C. Highly Selective Electrooxidation of Urea to Nitrogen on Copper/Nickel Boride Interface under Alkaline Condition. *Ind. Eng. Chem. Res.* **62**, 8736–8743 (2023).

38. Tatarchuk, S. W., Medvedev, J. J., Li, F., Tobolovskaya, Y. & Klinkova, A. Nickel-Catalyzed Urea Electrolysis: From Nitrite and Cyanate as Major Products to Nitrogen Evolution. *Angew. Chem. Int. Ed.* **61**, e202209839 (2022).

39. Li, J., et al. Deciphering and Suppressing Over-Oxidized Nitrogen in Nickel-Catalyzed Urea Electrolysis. *Angew. Chem. Int. Ed.* **60**, 26656–26662 (2021).

40. Wang, D., Vijapur, S. H., Wang, Y. & Botte, G. G. $NiCo_2O_4$ nanosheets grown on current collectors as binder-free electrodes for hydrogen production via urea electrolysis. *Int. J. Hydrogen Energy* **42**, 3987–3993 (2017).
