# Supplementary material for: Highly selective urea electrooxidation coupled with efficient hydrogen evolution
Source: Nat Commun. 2024 Jul 14;15:5918. doi: 10.1038/s41467-024-50343-8 (PMC11247087; doi:10.1038/s41467-024-50343-8)
Supplement: Supplementary file 3 — Description of Additional Supplementary Files [file 41467_2024_50343_MOESM3_ESM.pdf]

Description of Additional Supplementary Files

File Name: Supplementary Movie 1

Description: Chronoamperometry measurements of asymmetric Ni–O–Ti sites, Ni foam, and Ni_1@NC with the initial current density of 100 mA cm^{-2} in 1.0 M KOH containing 0.33 M urea.

File Name: Supplementary Data 1

Description: Optimized Ni–O–Ti and Ni–O–Ni structures in VASP POSCAR format.
