# Supplementary material for: Highly selective urea electrooxidation coupled with efficient hydrogen evolution
Source: Nat Commun. 2024 Jul 14;15:5918. doi: 10.1038/s41467-024-50343-8 (PMC11247087; doi:10.1038/s41467-024-50343-8)
Supplement: Supplementary file 4 — Supplementary Data 1 [file 41467_2024_50343_MOESM4_ESM.pdf]

Supplementary Data 1

Optimized Ni–O–Ti and Ni–O–Ni structures in VASP POSCAR format.

```
Ni–O–Ti structure
1.0
        20.4409999847         0.0000000000         0.0000000000
         5.2073195540        17.0316285534         0.0000000000
         0.0000000000         0.0000000000        28.7000007629
Ti    Ni    O    H
207    1    153    13
Direct
     0.151310000     0.619280000     0.413110000
     0.104230000     0.805490000     0.407880000
     0.031200000     0.953990000     0.461290000
     0.958320000     0.141730000     0.444280000
     0.889280000     0.308060000     0.440690000
     0.774660000     0.510750000     0.493140000
     0.768070000     0.639520000     0.422860000
     0.661490000     0.664880000     0.530070000
     0.651280000     0.968090000     0.438880000
     0.577950000     0.147680000     0.407650000
     0.538010000     0.310890000     0.426170000
     0.456180000     0.490400000     0.456430000
     0.388610000     0.662380000     0.440910000
     0.322100000     0.833400000     0.388820000
     0.235200000     0.044690000     0.423000000
     0.302340000     0.529190000     0.409100000
     0.238280000     0.755690000     0.449930000
     0.177000000     0.911160000     0.420230000
     0.100720000     0.067830000     0.477210000
     0.085580000     0.186310000     0.410980000
     0.983910000     0.343780000     0.396180000
     0.910470000     0.511760000     0.413940000
     0.820170000     0.780440000     0.424270000
     0.788960000     0.926680000     0.467990000
     0.741380000     0.076260000     0.432740000
     0.676020000     0.244780000     0.426630000
     0.608990000     0.409790000     0.457090000
     0.528670000     0.593620000     0.418790000
     0.459950000     0.774080000     0.424290000
     0.391200000     0.950930000     0.434730000
     0.219940000     0.211010000     0.445180000
```

43	0.099770000	0.356310000	0.418750000
44	0.053180000	0.523180000	0.422740000
45	0.940550000	0.673990000	0.392960000
46	0.940140000	0.838540000	0.430470000
47	0.882200000	0.008080000	0.444880000
48	0.806520000	0.196600000	0.487460000
49	0.755760000	0.366120000	0.461680000
50	0.663910000	0.566070000	0.431510000
51	0.598020000	0.722430000	0.448330000
52	0.545870000	0.870560000	0.401670000
53	0.351320000	0.119950000	0.408630000
54	0.305040000	0.297650000	0.424060000
55	0.198860000	0.446750000	0.428390000
56	0.487780000	0.044500000	0.438900000
57	0.453140000	0.210550000	0.459990000
58	0.395550000	0.378050000	0.440040000
59	0.217950000	0.547380000	0.332380000
60	0.164540000	0.701690000	0.325940000
61	0.102180000	0.893420000	0.324750000
62	0.058000000	0.046850000	0.372290000
63	0.959500000	0.225840000	0.343090000
64	0.883070000	0.375980000	0.335570000
65	0.863210000	0.550560000	0.333050000
66	0.809000000	0.717540000	0.340100000
67	0.729750000	0.893850000	0.359270000
68	0.674040000	0.050350000	0.354260000
69	0.594500000	0.235080000	0.337050000
70	0.533720000	0.437170000	0.363970000
71	0.440020000	0.574590000	0.356720000
72	0.415100000	0.719410000	0.342910000
73	0.323820000	0.980780000	0.338910000
74	0.402440000	0.453710000	0.344150000
75	0.300330000	0.663810000	0.359800000
76	0.227090000	0.808220000	0.335760000
77	0.192610000	0.986800000	0.333560000
78	0.113000000	0.145000000	0.321400000
79	0.082150000	0.297740000	0.329150000
80	0.007180000	0.479090000	0.334260000
81	0.943060000	0.648160000	0.301260000
82	0.885820000	0.816360000	0.340780000
83	0.827630000	0.976200000	0.362440000
84	0.730940000	0.174940000	0.348870000

85	0.662750000	0.344640000	0.348000000
86	0.632340000	0.509890000	0.338240000
87	0.564500000	0.699530000	0.352920000
88	0.447400000	0.856860000	0.338060000
89	0.245090000	0.138140000	0.341910000
90	0.206710000	0.308010000	0.349180000
91	0.140060000	0.452730000	0.332340000
92	0.067310000	0.606740000	0.338770000
93	0.028270000	0.767410000	0.331590000
94	0.967970000	0.949400000	0.360290000
95	0.880000000	0.101730000	0.360850000
96	0.800000000	0.274030000	0.382640000
97	0.769900000	0.461150000	0.367520000
98	0.713700000	0.605770000	0.333130000
99	0.671410000	0.766430000	0.362650000
100	0.437190000	0.010550000	0.351600000
101	0.349500000	0.226560000	0.333120000
102	0.301960000	0.393930000	0.347550000
103	0.582010000	0.977090000	0.344480000
104	0.487260000	0.126950000	0.346950000
105	0.449670000	0.283550000	0.361310000
106	0.154880000	0.613030000	0.251820000
107	0.108180000	0.786570000	0.252460000
108	0.018090000	0.966340000	0.263490000
109	0.977730000	0.115010000	0.294270000
110	0.890340000	0.302370000	0.251590000
111	0.819720000	0.469440000	0.267990000
112	0.789970000	0.634750000	0.263400000
113	0.717280000	0.807710000	0.279370000
114	0.664800000	0.964660000	0.270150000
115	0.592090000	0.115460000	0.282130000
116	0.522220000	0.326180000	0.284630000
117	0.470670000	0.484990000	0.265330000
118	0.396790000	0.640130000	0.268010000
119	0.336590000	0.838680000	0.287740000
120	0.253410000	0.032720000	0.263280000
121	0.310590000	0.556920000	0.281930000
122	0.256480000	0.711900000	0.261850000
123	0.199260000	0.900860000	0.256030000
124	0.110280000	0.045470000	0.261720000
125	0.039990000	0.234410000	0.255040000
126	0.978750000	0.386750000	0.270820000

127	0.922830000	0.539660000	0.243250000
128	0.860650000	0.750050000	0.252070000
129	0.804370000	0.906290000	0.277670000
130	0.755610000	0.073350000	0.285610000
131	0.683330000	0.249580000	0.267420000
132	0.600410000	0.425780000	0.272720000
133	0.534870000	0.602540000	0.287390000
134	0.490200000	0.757630000	0.267680000
135	0.403200000	0.945300000	0.258040000
136	0.196200000	0.202110000	0.265070000
137	0.137090000	0.352810000	0.256850000
138	0.064530000	0.522000000	0.257360000
139	0.025950000	0.688090000	0.246680000
140	0.959260000	0.858950000	0.274210000
141	0.884260000	0.009540000	0.276820000
142	0.830610000	0.190630000	0.291710000
143	0.756950000	0.362560000	0.291350000
144	0.688780000	0.519580000	0.257090000
145	0.639440000	0.701520000	0.270840000
146	0.571180000	0.851640000	0.300300000
147	0.352910000	0.102450000	0.278220000
148	0.281960000	0.290620000	0.261640000
149	0.230900000	0.441630000	0.260090000
150	0.501740000	0.028130000	0.268120000
151	0.451160000	0.210420000	0.267480000
152	0.383340000	0.361460000	0.273000000
153	0.194200000	0.519980000	0.522470000
154	0.507790000	0.360430000	0.530450000
155	0.872640000	0.319230000	0.545040000
156	0.151410000	0.172820000	0.524250000
157	0.451940000	0.061740000	0.537480000
158	0.816700000	0.941390000	0.592630000
159	0.143160000	0.871480000	0.529180000
160	0.136670000	0.720620000	0.516940000
161	0.423550000	0.552870000	0.564960000
162	0.934920000	0.421390000	0.491830000
163	0.167330000	0.357100000	0.512450000
164	0.473830000	0.289880000	0.623820000
165	0.784360000	0.124640000	0.607770000
166	0.219640000	0.921770000	0.610830000
167	0.415770000	0.731230000	0.534670000
168	0.884730000	0.631580000	0.498670000

169	0.557560000	0.882630000	0.506600000
170	0.875660000	0.814010000	0.519110000
171	0.591050000	0.422880000	0.596620000
172	0.049240000	0.225100000	0.602150000
173	0.629760000	0.106270000	0.608920000
174	0.929790000	0.905190000	0.636490000
175	0.332950000	0.665700000	0.632590000
176	0.700700000	0.528890000	0.611880000
177	0.998470000	0.417450000	0.594400000
178	0.313460000	0.336960000	0.650620000
179	0.619770000	0.259490000	0.639780000
180	0.937420000	0.601640000	0.655780000
181	0.653800000	0.803520000	0.602190000
182	0.294100000	0.601420000	0.499250000
183	0.692060000	0.435470000	0.537850000
184	0.027080000	0.278910000	0.497080000
185	0.330950000	0.218050000	0.508060000
186	0.630310000	0.125130000	0.500430000
187	0.946790000	0.067970000	0.624340000
188	0.292090000	0.875770000	0.489480000
189	0.267200000	0.744260000	0.553180000
190	0.564160000	0.592330000	0.519900000
191	0.059250000	0.499300000	0.525520000
192	0.349470000	0.412380000	0.554310000
193	0.683070000	0.258790000	0.530440000
194	0.968040000	0.148380000	0.539500000
195	0.329880000	0.070200000	0.495020000
196	0.703310000	0.791520000	0.479240000
197	0.008570000	0.652400000	0.485170000
198	0.685180000	0.959910000	0.552180000
199	0.010920000	0.831530000	0.552590000
200	0.198860000	0.622390000	0.622950000
201	0.495650000	0.552090000	0.689560000
202	0.230650000	0.234750000	0.609750000
203	0.084150000	0.934880000	0.664600000
204	0.130610000	0.777330000	0.617510000
205	0.562190000	0.688590000	0.634260000
206	0.845830000	0.500800000	0.592970000
207	0.167680000	0.435260000	0.606770000
208	0.760530000	0.313990000	0.633240000
209	0.477360000	0.865890000	0.593110000
210	0.836340000	0.766770000	0.655460000

211	0.029090000	0.706130000	0.660790000
212	0.325610000	0.026060000	0.622390000
213	0.134610000	0.084610000	0.608780000
214	0.788650000	0.682420000	0.580500000
215	0.376660000	0.805480000	0.653250000
216	0.383360000	0.161920000	0.603250000
217	0.547630000	0.989580000	0.617200000
218	0.364080000	0.486110000	0.663680000
219	0.515950000	0.407370000	0.703140000
220	0.300080000	0.416010000	0.418410000
221	0.698380000	0.345270000	0.412420000
222	0.985210000	0.238130000	0.423350000
223	0.278660000	0.213780000	0.382350000
224	0.701790000	0.017660000	0.485550000
225	0.068230000	0.931390000	0.392660000
226	0.328280000	0.752690000	0.494570000
227	0.295110000	0.643650000	0.432050000
228	0.604780000	0.487390000	0.401820000
229	0.007370000	0.444220000	0.403880000
230	0.366210000	0.298470000	0.478940000
231	0.709030000	0.168230000	0.477170000
232	0.008100000	0.062960000	0.495750000
233	0.257900000	0.943900000	0.387780000
234	0.639450000	0.804300000	0.426880000
235	0.953850000	0.591760000	0.443180000
236	0.732730000	0.871330000	0.431320000
237	0.050250000	0.636560000	0.413940000
238	0.184810000	0.532790000	0.589980000
239	0.500450000	0.465550000	0.557140000
240	0.839450000	0.325510000	0.605640000
241	0.251050000	0.167790000	0.510940000
242	0.362460000	0.067740000	0.561020000
243	0.911470000	0.874150000	0.569570000
244	0.138460000	0.883470000	0.597720000
245	0.132740000	0.683370000	0.577370000
246	0.472940000	0.629780000	0.553690000
247	0.909250000	0.412250000	0.562460000
248	0.172580000	0.332860000	0.576330000
249	0.548850000	0.333080000	0.597540000
250	0.799370000	0.202380000	0.641780000
251	0.137010000	0.072240000	0.540220000
252	0.440350000	0.819920000	0.537680000

253	0.844090000	0.578640000	0.548010000
254	0.514950000	0.959430000	0.555220000
255	0.855340000	0.722140000	0.545290000
256	0.292370000	0.507960000	0.529380000
257	0.763010000	0.318430000	0.525170000
258	0.974890000	0.257410000	0.554690000
259	0.427800000	0.175980000	0.543500000
260	0.604270000	0.055690000	0.552670000
261	0.038470000	0.913130000	0.526810000
262	0.358610000	0.750890000	0.591510000
263	0.326240000	0.633030000	0.560770000
264	0.673390000	0.549770000	0.503560000
265	0.023690000	0.396100000	0.525690000
266	0.304250000	0.244440000	0.571910000
267	0.625140000	0.194790000	0.551390000
268	0.898380000	0.127160000	0.573660000
269	0.292240000	0.984790000	0.477570000
270	0.668320000	0.662230000	0.395070000
271	0.973720000	0.586820000	0.530360000
272	0.652330000	0.894260000	0.506130000
273	0.029920000	0.738390000	0.517000000
274	0.548380000	0.476920000	0.646020000
275	0.156680000	0.188170000	0.594070000
276	0.515870000	0.646650000	0.677080000
277	0.118420000	0.833800000	0.678220000
278	0.518620000	0.913080000	0.643330000
279	0.222510000	0.551960000	0.455780000
280	0.537260000	0.518950000	0.475550000
281	0.844450000	0.394180000	0.487720000
282	0.202340000	0.327180000	0.423000000
283	0.544440000	0.118460000	0.468010000
284	0.880670000	0.102700000	0.472270000
285	0.244510000	0.869280000	0.549240000
286	0.152110000	0.714400000	0.448160000
287	0.476530000	0.681980000	0.468290000
288	0.840120000	0.555040000	0.459710000
289	0.232460000	0.409650000	0.497120000
290	0.506390000	0.279560000	0.484770000
291	0.895650000	0.228530000	0.495690000
292	0.164830000	0.129510000	0.454300000
293	0.522270000	0.819760000	0.463830000
294	0.916810000	0.742830000	0.460190000

295	0.562790000	0.950480000	0.448060000
296	0.887180000	0.911840000	0.480370000
297	0.418010000	0.586860000	0.628370000
298	0.695210000	0.421660000	0.611920000
299	0.395270000	0.247420000	0.640990000
300	0.553650000	0.198550000	0.632980000
301	0.001640000	0.959690000	0.626400000
302	0.475030000	0.776960000	0.627340000
303	0.356530000	0.721610000	0.680760000
304	0.721660000	0.621970000	0.586200000
305	0.066730000	0.470230000	0.591920000
306	0.379770000	0.473090000	0.597330000
307	0.680400000	0.297120000	0.600530000
308	0.953330000	0.107200000	0.682090000
309	0.388650000	0.927480000	0.604800000
310	0.752040000	0.776890000	0.620790000
311	0.841840000	0.650160000	0.635550000
312	0.834770000	0.873070000	0.644340000
313	0.022580000	0.799850000	0.619300000
314	0.417160000	0.425250000	0.499400000
315	0.746900000	0.470200000	0.433720000
316	0.128370000	0.276270000	0.483510000
317	0.400450000	0.127850000	0.468840000
318	0.729940000	0.029710000	0.587590000
319	0.130150000	0.952290000	0.479030000
320	0.372260000	0.850070000	0.446380000
321	0.395990000	0.578350000	0.492260000
322	0.770230000	0.474000000	0.559000000
323	0.108030000	0.440690000	0.467200000
324	0.433160000	0.326100000	0.565190000
325	0.769190000	0.173600000	0.550210000
326	0.026140000	0.110990000	0.594730000
327	0.435340000	0.987650000	0.484300000
328	0.792580000	0.708850000	0.469240000
329	0.102750000	0.581370000	0.504240000
330	0.783850000	0.894220000	0.537310000
331	0.026560000	0.842940000	0.448420000
332	0.289370000	0.583120000	0.653870000
333	0.611390000	0.515240000	0.567880000
334	0.031770000	0.327710000	0.626150000
335	0.300630000	0.141370000	0.632140000
336	0.862720000	0.019360000	0.621740000

337	0.145440000	0.993590000	0.650220000
338	0.232840000	0.721640000	0.616040000
339	0.583480000	0.665410000	0.569560000
340	0.932030000	0.503230000	0.627230000
341	0.279620000	0.395070000	0.596480000
342	0.616550000	0.374490000	0.525270000
343	0.239160000	0.020610000	0.600920000
344	0.574450000	0.818330000	0.561620000
345	0.645810000	0.912820000	0.600370000
346	0.028440000	0.598040000	0.658440000
347	0.930980000	0.716170000	0.642500000
348	0.705260000	0.748730000	0.543940000
349	0.284970000	0.870140000	0.649980000
350	0.616890000	0.754260000	0.646880000
351	0.335400000	0.008310000	0.684320000
352	0.041230000	0.983510000	0.719840000
353	0.010070000	0.742830000	0.719350000
354	0.601850000	0.042570000	0.648840000
355	0.691850000	0.154050000	0.638370000
356	0.144420000	0.464950000	0.667240000
357	0.451110000	0.366750000	0.666150000
358	0.086820000	0.160120000	0.660130000
359	0.916760000	0.601960000	0.719260000
360	0.936900000	0.892610000	0.702820000
361	0.405890000	0.859380000	0.701590000
362	0.129560000	0.676110000	0.662450000
363	0.599810000	0.318400000	0.689510000
364	0.827700000	0.757310000	0.718050000
365	0.746170000	0.341160000	0.694820000
366	0.238730000	0.300050000	0.659290000
367	0.643100000	0.593550000	0.659070000
368	0.787670000	0.503770000	0.649870000
369	0.465390000	0.076050000	0.611950000
370	0.419450000	0.509090000	0.707250000
371	0.545540000	0.497160000	0.734860000
372	0.315630000	0.420250000	0.687180000
373	0.359920000	0.954040000	0.696220000
374	0.019040000	0.952090000	0.738560000
375	0.984950000	0.800670000	0.719640000
376	0.104350000	0.512060000	0.671330000
377	0.052140000	0.144650000	0.678310000
378	0.982560000	0.064730000	0.703490000

379	0.893780000	0.895580000	0.718470000
380	0.954400000	0.597870000	0.740850000
381	0.854650000	0.704940000	0.729580000
382	0.701260000	0.344870000	0.709100000
383	0.625740000	0.569580000	0.685280000
384	0.788280000	0.536040000	0.677040000
385	0.452670000	0.863210000	0.701180000
386			

Ni–O–Ni structure

```
1.0
20.4409999847    0.0000000000    0.0000000000
5.2073195540    17.0316285534    0.0000000000
0.0000000000    0.0000000000    28.7000007629
Ti Ni O H
206 2 152 13
Direct
0.150570000 0.620370000 0.412970000
0.103500000 0.806390000 0.407910000
0.030450000 0.955020000 0.461300000
0.959730000 0.141600000 0.444330000
0.888810000 0.308540000 0.439720000
0.773750000 0.507660000 0.494310000
0.768100000 0.635950000 0.422660000
0.677060000 0.652050000 0.516440000
0.648970000 0.965680000 0.439030000
0.577360000 0.147250000 0.408160000
0.538310000 0.308000000 0.428620000
0.453580000 0.492260000 0.455290000
0.390590000 0.662320000 0.440060000
0.321680000 0.834330000 0.388360000
0.235650000 0.045440000 0.423710000
0.302820000 0.530110000 0.409320000
0.237790000 0.756570000 0.449080000
0.176860000 0.912000000 0.420210000
0.101230000 0.069110000 0.477750000
0.085970000 0.187280000 0.411530000
0.984280000 0.343890000 0.396180000
0.910790000 0.511790000 0.413250000
0.818920000 0.778720000 0.424930000
0.788370000 0.925860000 0.468270000
0.742290000 0.074070000 0.432360000
0.675850000 0.243620000 0.425730000
0.609360000 0.407940000 0.455380000
0.529220000 0.590860000 0.416790000
0.459610000 0.773880000 0.425550000
0.391150000 0.952680000 0.435360000
0.220600000 0.212110000 0.446320000
0.100260000 0.356240000 0.418670000
0.052890000 0.523490000 0.422520000
0.939900000 0.673700000 0.393290000
```

429	0.939450000	0.837770000	0.431030000
430	0.881310000	0.009260000	0.445620000
431	0.809720000	0.191530000	0.483650000
432	0.755060000	0.365900000	0.461300000
433	0.663060000	0.564300000	0.428930000
434	0.602450000	0.719030000	0.446730000
435	0.545790000	0.872430000	0.403780000
436	0.351980000	0.120240000	0.408500000
437	0.306350000	0.298100000	0.424480000
438	0.198870000	0.447630000	0.427800000
439	0.488480000	0.043660000	0.440020000
440	0.455040000	0.210970000	0.463590000
441	0.394850000	0.380370000	0.441900000
442	0.217950000	0.547380000	0.332380000
443	0.164540000	0.701690000	0.325940000
444	0.102180000	0.893420000	0.324750000
445	0.058030000	0.047110000	0.372190000
446	0.959500000	0.225840000	0.343090000
447	0.883070000	0.375980000	0.335570000
448	0.863210000	0.550560000	0.333050000
449	0.809000000	0.717540000	0.340100000
450	0.730260000	0.894490000	0.359760000
451	0.673820000	0.050650000	0.354290000
452	0.594500000	0.235080000	0.337050000
453	0.533340000	0.434610000	0.363350000
454	0.439290000	0.573890000	0.356720000
455	0.415100000	0.719410000	0.342910000
456	0.323820000	0.980780000	0.338910000
457	0.402440000	0.453710000	0.344150000
458	0.300010000	0.664520000	0.359750000
459	0.227090000	0.808220000	0.335760000
460	0.192610000	0.986800000	0.333560000
461	0.113000000	0.145000000	0.321400000
462	0.082150000	0.297740000	0.329150000
463	0.007180000	0.479090000	0.334260000
464	0.943060000	0.648160000	0.301260000
465	0.885820000	0.816360000	0.340780000
466	0.828260000	0.975330000	0.362520000
467	0.730940000	0.174940000	0.348870000
468	0.662750000	0.344640000	0.348000000
469	0.632340000	0.509890000	0.338240000
470	0.564840000	0.700010000	0.351210000

471	0.447400000	0.856860000	0.338060000
472	0.245090000	0.138140000	0.341910000
473	0.206980000	0.307830000	0.349680000
474	0.140060000	0.452730000	0.332340000
475	0.067310000	0.606740000	0.338770000
476	0.028270000	0.767410000	0.331590000
477	0.967920000	0.949350000	0.360340000
478	0.879620000	0.101720000	0.361040000
479	0.799470000	0.274150000	0.382030000
480	0.770330000	0.461200000	0.367210000
481	0.713700000	0.605770000	0.333130000
482	0.671540000	0.767580000	0.362550000
483	0.438320000	0.009400000	0.352450000
484	0.349500000	0.226560000	0.333120000
485	0.301960000	0.393930000	0.347550000
486	0.582010000	0.977090000	0.344480000
487	0.487260000	0.126950000	0.346950000
488	0.449490000	0.283500000	0.362620000
489	0.154880000	0.613030000	0.251820000
490	0.108180000	0.786570000	0.252460000
491	0.018090000	0.966340000	0.263490000
492	0.977730000	0.115010000	0.294270000
493	0.890340000	0.302370000	0.251590000
494	0.819720000	0.469440000	0.267990000
495	0.789970000	0.634750000	0.263400000
496	0.717280000	0.807710000	0.279370000
497	0.664800000	0.964660000	0.270150000
498	0.592090000	0.115460000	0.282130000
499	0.522220000	0.326180000	0.284630000
500	0.470670000	0.484990000	0.265330000
501	0.396790000	0.640130000	0.268010000
502	0.336590000	0.838680000	0.287740000
503	0.253410000	0.032720000	0.263280000
504	0.310590000	0.556920000	0.281930000
505	0.256480000	0.711900000	0.261850000
506	0.199260000	0.900860000	0.256030000
507	0.110280000	0.045470000	0.261720000
508	0.039990000	0.234410000	0.255040000
509	0.978750000	0.386750000	0.270820000
510	0.922830000	0.539660000	0.243250000
511	0.860650000	0.750050000	0.252070000
512	0.804370000	0.906290000	0.277670000

513	0.755610000	0.073350000	0.285610000
514	0.683330000	0.249580000	0.267420000
515	0.600410000	0.425780000	0.272720000
516	0.534870000	0.602540000	0.287390000
517	0.490200000	0.757630000	0.267680000
518	0.403200000	0.945300000	0.258040000
519	0.196200000	0.202110000	0.265070000
520	0.137090000	0.352810000	0.256850000
521	0.064530000	0.522000000	0.257360000
522	0.025950000	0.688090000	0.246680000
523	0.959260000	0.858950000	0.274210000
524	0.884260000	0.009540000	0.276820000
525	0.830610000	0.190630000	0.291710000
526	0.756950000	0.362560000	0.291350000
527	0.688780000	0.519580000	0.257090000
528	0.639440000	0.701520000	0.270840000
529	0.571180000	0.851640000	0.300300000
530	0.352910000	0.102450000	0.278220000
531	0.281960000	0.290620000	0.261640000
532	0.230900000	0.441630000	0.260090000
533	0.501740000	0.028130000	0.268120000
534	0.451160000	0.210420000	0.267480000
535	0.383340000	0.361460000	0.273000000
536	0.193900000	0.521380000	0.522950000
537	0.517450000	0.365760000	0.529470000
538	0.870900000	0.324090000	0.543990000
539	0.151120000	0.175750000	0.525300000
540	0.453930000	0.059440000	0.540280000
541	0.819570000	0.941900000	0.592070000
542	0.141000000	0.873230000	0.528750000
543	0.136530000	0.721620000	0.516590000
544	0.426610000	0.561920000	0.565670000
545	0.930320000	0.427470000	0.492390000
546	0.168000000	0.358700000	0.512010000
547	0.480390000	0.272790000	0.629870000
548	0.796360000	0.134370000	0.607500000
549	0.216070000	0.924770000	0.610420000
550	0.408550000	0.739400000	0.537040000
551	0.881400000	0.632260000	0.499790000
552	0.557980000	0.872710000	0.509950000
553	0.874510000	0.811870000	0.520260000
554	0.582730000	0.427370000	0.609210000

555	0.046330000	0.230390000	0.601810000
556	0.637750000	0.093470000	0.611650000
557	0.931340000	0.904810000	0.636210000
558	0.330940000	0.680550000	0.635450000
559	0.658480000	0.555600000	0.618510000
560	0.993600000	0.423210000	0.593410000
561	0.326000000	0.332250000	0.635700000
562	0.629090000	0.251320000	0.628700000
563	0.941060000	0.602860000	0.655390000
564	0.659550000	0.800730000	0.599770000
565	0.297170000	0.604180000	0.499160000
566	0.687120000	0.436620000	0.540550000
567	0.026320000	0.281150000	0.496980000
568	0.331750000	0.220090000	0.510220000
569	0.631090000	0.126110000	0.500540000
570	0.952110000	0.071020000	0.624990000
571	0.290140000	0.879130000	0.488340000
572	0.264800000	0.748490000	0.552020000
573	0.561680000	0.594910000	0.515540000
574	0.059550000	0.499180000	0.525900000
575	0.351470000	0.419290000	0.554270000
576	0.687390000	0.258730000	0.527510000
577	0.964080000	0.150470000	0.538600000
578	0.331330000	0.072010000	0.495990000
579	0.703970000	0.788500000	0.479000000
580	0.007470000	0.650090000	0.485950000
581	0.682250000	0.958840000	0.549830000
582	0.011090000	0.830620000	0.551810000
583	0.200520000	0.625530000	0.622860000
584	0.230770000	0.238650000	0.609730000
585	0.083380000	0.936200000	0.667050000
586	0.130330000	0.779950000	0.617370000
587	0.558450000	0.715630000	0.612920000
588	0.831630000	0.504530000	0.598570000
589	0.169710000	0.436550000	0.606540000
590	0.758180000	0.325410000	0.633770000
591	0.471550000	0.873510000	0.591080000
592	0.838210000	0.767910000	0.655000000
593	0.031310000	0.707550000	0.660990000
594	0.321560000	0.026680000	0.623180000
595	0.131830000	0.088710000	0.609520000
596	0.787960000	0.673350000	0.585520000

597	0.373050000	0.824860000	0.653440000
598	0.383540000	0.156870000	0.604470000
599	0.548150000	0.985030000	0.619620000
600	0.364270000	0.495730000	0.660580000
601	0.501250000	0.557990000	0.654080000
602	0.527530000	0.399360000	0.684120000
603	0.300480000	0.416610000	0.418370000
604	0.698330000	0.344250000	0.411870000
605	0.985210000	0.238300000	0.423290000
606	0.278880000	0.213770000	0.382840000
607	0.700350000	0.016120000	0.485210000
608	0.067930000	0.931820000	0.392710000
609	0.326660000	0.755440000	0.493270000
610	0.296150000	0.644810000	0.432000000
611	0.604320000	0.485460000	0.401020000
612	0.007480000	0.444350000	0.403550000
613	0.366630000	0.299180000	0.480400000
614	0.710720000	0.166960000	0.475790000
615	0.009760000	0.064110000	0.496500000
616	0.257980000	0.945120000	0.387820000
617	0.639670000	0.805040000	0.426720000
618	0.952930000	0.591290000	0.443340000
619	0.731980000	0.870790000	0.431680000
620	0.049580000	0.637020000	0.414610000
621	0.183480000	0.537470000	0.590430000
622	0.501150000	0.468210000	0.562870000
623	0.836510000	0.326820000	0.604040000
624	0.251910000	0.169590000	0.513220000
625	0.364400000	0.061400000	0.562830000
626	0.912490000	0.873420000	0.569030000
627	0.136030000	0.885890000	0.597240000
628	0.133430000	0.685820000	0.577530000
629	0.476280000	0.642610000	0.552490000
630	0.903080000	0.419410000	0.562760000
631	0.171880000	0.334200000	0.575570000
632	0.547060000	0.327670000	0.593770000
633	0.798460000	0.212820000	0.644980000
634	0.135780000	0.075850000	0.540770000
635	0.434890000	0.827420000	0.536320000
636	0.830440000	0.583840000	0.544400000
637	0.515280000	0.954660000	0.556710000
638	0.854920000	0.720990000	0.545910000

639	0.291110000	0.513080000	0.528390000
640	0.761380000	0.321460000	0.523960000
641	0.972080000	0.259920000	0.554080000
642	0.427530000	0.174730000	0.544540000
643	0.605210000	0.056050000	0.553380000
644	0.036420000	0.914240000	0.526580000
645	0.351220000	0.762970000	0.591000000
646	0.326690000	0.640320000	0.559050000
647	0.669020000	0.539190000	0.500570000
648	0.019330000	0.398650000	0.524990000
649	0.307860000	0.249590000	0.575050000
650	0.629920000	0.196290000	0.549980000
651	0.895160000	0.134950000	0.578520000
652	0.293030000	0.987030000	0.477780000
653	0.668930000	0.661740000	0.393440000
654	0.971030000	0.580820000	0.529720000
655	0.652770000	0.886660000	0.508730000
656	0.029910000	0.736740000	0.516940000
657	0.575630000	0.472340000	0.668540000
658	0.153950000	0.192130000	0.594800000
659	0.568390000	0.618390000	0.641210000
660	0.119360000	0.834530000	0.678540000
661	0.513090000	0.912850000	0.645090000
662	0.221650000	0.553180000	0.454810000
663	0.538420000	0.515880000	0.475750000
664	0.843670000	0.394130000	0.486070000
665	0.203000000	0.327640000	0.423540000
666	0.545010000	0.116630000	0.469100000
667	0.886610000	0.099070000	0.476580000
668	0.241360000	0.873510000	0.547710000
669	0.151780000	0.715200000	0.447960000
670	0.483040000	0.677070000	0.465430000
671	0.842360000	0.547560000	0.460330000
672	0.231000000	0.413460000	0.495580000
673	0.505840000	0.280920000	0.489480000
674	0.894570000	0.230280000	0.494970000
675	0.165630000	0.130780000	0.455190000
676	0.524590000	0.814180000	0.464460000
677	0.915670000	0.741780000	0.460910000
678	0.561000000	0.948570000	0.453900000
679	0.886270000	0.911100000	0.479990000
680	0.417620000	0.610110000	0.626390000

681	0.685710000	0.428610000	0.612220000
682	0.401590000	0.231080000	0.646220000
683	0.563670000	0.187580000	0.629820000
684	0.002050000	0.961120000	0.628370000
685	0.468920000	0.780030000	0.628620000
686	0.353420000	0.737420000	0.681440000
687	0.732650000	0.602380000	0.603370000
688	0.067240000	0.469440000	0.591480000
689	0.378870000	0.487590000	0.595690000
690	0.688280000	0.294320000	0.594450000
691	0.958820000	0.108630000	0.683280000
692	0.378370000	0.921890000	0.609940000
693	0.753200000	0.778580000	0.620250000
694	0.844930000	0.654060000	0.637610000
695	0.836350000	0.874680000	0.643850000
696	0.023360000	0.802210000	0.619080000
697	0.421380000	0.423500000	0.503560000
698	0.746060000	0.469850000	0.433210000
699	0.127840000	0.277840000	0.484030000
700	0.399400000	0.131020000	0.468100000
701	0.728210000	0.016040000	0.591090000
702	0.129040000	0.953670000	0.478770000
703	0.372020000	0.851510000	0.445410000
704	0.399780000	0.580740000	0.494000000
705	0.767630000	0.470330000	0.557660000
706	0.106780000	0.441090000	0.467030000
707	0.407590000	0.333830000	0.587450000
708	0.774310000	0.173890000	0.547240000
709	0.024710000	0.112010000	0.593900000
710	0.433690000	0.991090000	0.485380000
711	0.792030000	0.703840000	0.469820000
712	0.102260000	0.581290000	0.504770000
713	0.784140000	0.894460000	0.537580000
714	0.025760000	0.843570000	0.448420000
715	0.291050000	0.593550000	0.656910000
716	0.604230000	0.515700000	0.569060000
717	0.026540000	0.332800000	0.625600000
718	0.296060000	0.142280000	0.629790000
719	0.864860000	0.019540000	0.619910000
720	0.141320000	0.997130000	0.650070000
721	0.232060000	0.726780000	0.615390000
722	0.606200000	0.653740000	0.560420000

723	0.930980000	0.511780000	0.623130000
724	0.274380000	0.404550000	0.589780000
725	0.615910000	0.374180000	0.524060000
726	0.234670000	0.024370000	0.601490000
727	0.568140000	0.806760000	0.567050000
728	0.642060000	0.910760000	0.598820000
729	0.032210000	0.599630000	0.657440000
730	0.933840000	0.718760000	0.642680000
731	0.706710000	0.737770000	0.544750000
732	0.275940000	0.877130000	0.653170000
733	0.625010000	0.747960000	0.644320000
734	0.332330000	0.017880000	0.685880000
735	0.040490000	0.982530000	0.722630000
736	0.013110000	0.742340000	0.720070000
737	0.605060000	0.034030000	0.651990000
738	0.700900000	0.147910000	0.633430000
739	0.146480000	0.465820000	0.666980000
740	0.479810000	0.324890000	0.681010000
741	0.081940000	0.164720000	0.659810000
742	0.922260000	0.602620000	0.718900000
743	0.937690000	0.891510000	0.702600000
744	0.405000000	0.869890000	0.704020000
745	0.131890000	0.678170000	0.662570000
746	0.615320000	0.318910000	0.675600000
747	0.830430000	0.759740000	0.717640000
748	0.756280000	0.359300000	0.694500000
749	0.246710000	0.305380000	0.654630000
750	0.689550000	0.534250000	0.680190000
751	0.813760000	0.478580000	0.656800000
752	0.468360000	0.072840000	0.612210000
753	0.445610000	0.491280000	0.686480000
754	0.335880000	0.408740000	0.675390000
755	0.357080000	0.965480000	0.699530000
756	0.017000000	0.951260000	0.740430000
757	0.986680000	0.799600000	0.720460000
758	0.106470000	0.513270000	0.670750000
759	0.046350000	0.148860000	0.677030000
760	0.985130000	0.065400000	0.705340000
761	0.894380000	0.893400000	0.717790000
762	0.959860000	0.602700000	0.739920000
763	0.855480000	0.706560000	0.729290000
764	0.795250000	0.377240000	0.703840000

765	0.670690000	0.495020000	0.694740000
766	0.766860000	0.501610000	0.672570000
767	0.451010000	0.876030000	0.700780000
